# M1 Microglia-derived Exosomes Promote Activation of Resting Microglia and Amplifies Proangiogenic Effects through Irf1/miR-155-5p/Socs1 Axis in the Retina

**DOI:** 10.7150/ijbs.79784

**Published:** 2023-03-21

**Authors:** Xi Chen, Xiao Wang, Zedu Cui, Qian Luo, Zihua Jiang, Yuke Huang, Jingyi Jiang, Jin Qiu, Yan Li, Keming Yu, Jing Zhuang

**Affiliations:** 1 State Key Laboratory of Ophthalmology, Zhongshan Ophthalmic Center, Sun Yat-sen University, No.7 Jinsui Road, Tianhe District, Guangzhou, 510060, China; 2 Guangdong Provincial Key Laboratory of Ophthalmology and Visual Science, Guangzhou, 510060, China; 3 Guangdong Provincial Clinical Research Center for Ocular Diseases, Guangzhou, 510060, China

**Keywords:** microglia, exosomes, inflammation, neovascularization, retina

## Abstract

Activation of microglia plays a key role in the development of neovascular retinal diseases. Therefore, it is essential to reveal its pathophysiological and molecular mechanisms to interfere with disease progression. Here a publicly available single-cell RNA sequencing dataset is used to identify that intercellular communications from M1 microglia toward M0 microglia are increased in the retinal angiogenesis model via exosomes. Moreover, the results both *in vitro* and *in vivo* demonstrate that M1 microglia-derived exosomes promote the activation and enhance the proangiogenic ability of resting microglia. Based on miRNA sequencing of exosomes combined with gene interference, further results show that activated microglia-derived exosomes promoted microglial activation by transmitting polarized signals to M0 microglia via miR-155-5p. Subsequently, miR-155-5p suppresses Socs1 and activates the NFκB pathway, which ultimately causes the inflammatory cascade and amplifies the proangiogenic effect. In addition, upregulated Irf1 drives the expression of miR-155-5p in activated microglia, thus leading to an increase in the tendency of miR-155-5p to be encapsulated by exosomes. Thus, this study elucidates the critical role of intercellular communication among various types of microglia in the complex retinal microenvironment during angiogenesis, and contributes to the novel, targeted, and potential therapeutic strategies for clinical retinal neovascularization.

## Introduction

Pathological retinal neovascularization is the most common cause of irreversible blindness in individuals of all age groups, including newborns (retinopathy of prematurity), middle-aged adults (proliferative diabetic retinopathy), and the elderly (age-related macular degeneration) 1,2. In the one hand, increased endothelial sprouting and proliferation are critical cellular events during pathological neovascularization 3. On the other hand, microglia, the retinal resident macrophages, play pivotal roles during vascular development and angiogenesis in the retina 4. Therefore, deciphering the mechanisms underlying these early cellular events is vital to developing novel therapeutic approaches for the early intervention of these vision-threatening diseases.

Microglia in a resting homeostatic state without activation are often referred to as M0 (resting) microglia. When retinal homeostasis is disrupted due to various physiological and pathological factors such as injury, hypoxia, neuroinflammation, degeneration, and/or aging, microglia undergo several specific activation processes 5-7. Microglial activation can be classified into two major phenotypes defined as "classical activation" (also termed the M1 phenotype, proinflammatory) and "alternative activation” (M2 phenotype, anti-inflammatory) 8. Increasing evidence indicates that M1 microglial activation aggravates aberrant retinal neovascularization. Many studies have demonstrated the direct effects of activated microglia on endothelial cells during retinal neovascularization. For example, Sun *et al.* and Zhu *et al.* noted that microglia become activated and release proangiogenic mediators to stimulate retinal neovascularization in a mouse model of oxygen-induced retinopathy 9,10. In addition, under pathological stimulation, microglia transform rapidly into an amoeboid morphology and can rapidly accumulate at the injury site. Liu *et al.* reported that Iba1-positive microglia were observed at lesions from Day 1 to Day 7 and peaked at Day 2 in a laser-induced choroidal neovascularization (CNV) model 11. Between Days 7 and 14, microglial numbers progressively subsided, and neovascularization normally occurred on Day 14. Therefore, the accumulation of microglia at the injury site is transient and prior to the onset of retinal angiogenesis. However, how this proangiogenic "microglial army" was recruited and reacted in the pathological process of retinal neovascularization was not well defined.

In addition, many studies indicate that retinal inflammation is critical in the pathogenesis of neovascular retinopathies 12. Microglia are constantly serving their environment and closely interacting with surrounding cells. Prolonged activation of microglial inflammatory responses can lead to an increase in pathological retinal neovascularization. For example, Connor *et al.* reported that retinal microglia highly expressing inflammatory cytokines were involved in retinal angiogenesis, acting partly through the regulation of NF-κB-driven TNFα production 13. Given that microglia can propagate inflammatory signals, it is worthwhile to investigate whether M1 microglia can initiate an inflammatory response in other microglia, thus triggering a domino effect during neovascularization development 14,15. Specifically, it is unclear whether M1 microglia can activate resting microglia to stimulate an inflammatory cascade and enhance the proangiogenic effect. Moreover, the effective mediator that transmits signals to resting microglia in this process remains unknown. To date, these perplexing issues have not been convincingly resolved.

Upon activation, microglia secrete a variety of cytokines and chemokines, such as IL-1β, TNF-α, and CCL2, which can modulate neuroinflammation 16,17. However, the knowledge regarding the underlying mechanisms of microglia activation is limited, which may hinder the development of new therapeutic strategies. To address this issue, a growing body of literature demonstrates that exosomes play an essential role in intercellular communication and cell signal transduction 18,19. Exosomes are a class of extracellular vehicles (30-150 nm diameter lipid-bilayered vesicles) containing microRNAs (miRNAs), mRNAs, and proteins 20. Several studies have recently demonstrated that exosomes derived from damaged cells participate in many pathological situations 21,22. For example, An *et al.* found that exosomes from the injured brain could induce nearby neurons and astrocytes to migrate toward the injury core 21. Awdishu *et al.* noted that exosomes might communicate injury signals to recruit macrophage infiltration and promote inflammation after acute kidney injury 22. Moreover, similar biological processes exist in some retinopathies. Liu *et al.* found that exosomes isolated from vitreous humor of patients with proliferative diabetic retinopathy could promote angiogenesis of endothelial cells 23. Despite extensive research efforts, there is no evidence directly addressing the role of M1 microglia-derived exosomes in microglia activation, and whether these exosomes can contribute to the inflammatory cascade and amplifies the proangiogenic effect remains to be investigated.

Thus, focusing on the perplexities above, we utilized a series of experiments and bioinformatic analyses to elucidate M1 microglial exosome-associated cellular and molecular mechanisms in retinal neovascularization. A publicly available single-cell RNA sequencing dataset of the oxygen-induced retinopathy (OIR) model was reanalyzed to clarify potential links among various clusters of microglia and vascular endothelial cells. Based on* in vitro* and *in vivo* experiments, we found that M1 microglial exosomes polarized resting microglia toward the M1 proinflammatory phenotype and enhanced the proangiogenic potential of microglia. Evidence from exosomal miRNA-seq and gene interference showed that exosomal miR-155-5p acted as a mediator to spread the activated signal among resting microglia, and the functions of miR-155-5p correlated with the regulation of the Socs1/NFκB signaling pathway. We also elucidated the mechanism of the upregulated miR-155-5p using transcriptomics analyses and molecular experiments: the enriched upstream transcription factor Irf1 in M1-activated microglia. Based on our findings, we provide novel insights into disease development and new ideas for preventing and treating retinal neovascularization. Moreover, a novel mouse model of retinal neovascularization could be induced by intravitreal injection of activated microglia-derived exosomes.

## Methods

### Animals and ethics statement

Adult and newborn (male and female) C57BL/6J mice were obtained from the Ophthalmic Animal Laboratory, Zhongshan Ophthalmic Center, Sun Yat-sen University (Guangzhou, China). All animal experiments at the Zhongshan Ophthalmic Center received approval [no. SYXK (YUE) 2017-093] from the Institutional Animal Care and Use Committee, which also oversaw their compliance with the ARVO Statement for the Use of Animals in Ophthalmic and Vision Research. The animals were kept on a 12/12 hours light-dark cycle with free access to food and water.

### Data analysis for single-cell sequencing

Download the publicly available single-cell RNA sequencing dataset (accession no. GSE152928) from the Gene Expression Omnibus Dataset 24. Seurat (version 3.0) were used to examine single-cell data. For each dataset, cells that expressed less than 200 genes, had less than 80% UMI generated from protein-coding genes, and had more than 15% UMI derived from mitochondrial genes were eliminated. Moreover, the data were standardized by log normalizing gene content. Unsupervised clustering was accomplished using t-distributed stochastic neighbor embedding (t-SNE). All clusters and subclusters were manually annotated using known cell-type marker genes. CellPhoneDB (version 2.1.1) was used to accomplish cell cross-talk interaction 25. The average ligand and receptor expression in a certain cell type is represented by the mean value, which is computed using the percentage of cells expressing the relevant gene and the gene expression mean.

### Culture of primary retinal microglial cells

Primary retinal microglial cell culture was carried out using normal procedures with a few modifications 26,27. After anesthesia with 1% Pentobarbital Sodium and sacrifice, the eyes of postnatal Day 1-3 C57BL/6J mice were collected. After the choroid, sclera, and vitreous were removed, the retina was carefully dissected and trypsinized to form a single-cell suspension. Cells from two retinas were grown in DMEM/F12 supplemented with 20% fetal bovine serum (FBS), 100 U/mL penicillin, and 100 g/mL streptomycin in a 75 cm^2^ culture flask (1 × 10^6^ cells/mL). After 14 days of growth, retinal microglial cells were isolated from mixed glial cells by shaking the flask on a shaker at 220 rpm for three hours and then purified using a differential adhesion approach for 30 minutes. Microglia were planted in 6-well plates at a density of 5 × 10^5^ cells/mL for 48 hours.

### Culture of cell lines

The National Infrastructure of Cell Line Resource provided the BV2 microglial cell lines (Beijing, China). ScienCell provided us with human umbilical vein endothelial cells (HUVECs). After 48 hours of sufficient exosome uptake and metabolism in HUVECs, the media was changed with serum-free DMEM/F12. HUVECs were treated with various exosomes and PBS as controls after additional 24 hours in serum-free media. BV2 cells were grown in DMEM supplemented with 10% fetal bovine serum (Gibco, CA, USA). BV2 cells were treated with Lipopolysaccharide (LPS, 100 ng/mL, L6529, Sigma Aldrich) for 24 hours, and washed five times with PBS to ensure the complete removal of residual LPS. HUVECs were grown in ECM medium (ScienCell) supplemented with 1% ECGS and 5% fetal bovine serum. The cells were grown in a humidified environment with a mixture of 1% O_2_, 5% CO_2_, and 94% N_2_ at 37 °C.

### Isolation, characterization, and detection of exosomes

Ultracentrifugation was used to separate exosomes from the BV2-derived conditioned medium. The fetal bovine serum was depleted of exosomes by ultracentrifugation at 1.1×10^5^ g overnight at 4 °C prior to use. After five PBS washes to ensure the complete removal of residual LPS, the conditioned medium (containing exosome-depleted FBS) was added. After 48 hours, the conditioned medium was collected and centrifuged at 1000g for 5 minutes at 4 °C, followed by 10^4^ g for 30 minutes at 4 °C. The supernatants were ultracentrifuged for 90 minutes at 4 °C at 100,000g. Phosphate-buffered saline (PBS) was used to rinse the exosomes (PBS), then centrifuged at 100,000g for 90 minutes at 4 °C before being resuspended in PBS. Nanoparticle Tracking Analysis (NTA) was used to evaluate the size distribution of exosomes, and the BCA Protein Assay kit were used to determine the number of exosomes (Beyotime). For transmission electron microscopy, exosomes were fixed with 2% paraformaldehyde and placed on 200-mesh Formvar-coated grids. The grids were then stained for 2 minutes with 2% phosphotungstic acid and examined under a transmission electron microscope (FEI TECNAI spirit G2, USA). PKH26 membrane dye was used to fluorescently mark exosomes (Sigma, St. Louis, MO). Labeled exosomes were washed in PBS for 10 ml, ultracentrifuged, and resuspended in PBS. 10 μg of exosomes were incubated with 1 × 10^4^ recipient cells for cell treatment.

### Western blot

The tissues, cells and exosomes were lysed with RIPA buffer (50 mM Tris-HCl, pH 8.0, with 150 mM sodium chloride, 1.0% Igepal CA-630 (NP-40), 0.5% sodium deoxycholate, and 0.1% sodium dodecyl sulfate). Total protein was extracted after centrifuging the tubes at 4 °C for 15 minutes at maximum speed to remove debris. The following primary antibodies were used: β-Tubulin (CST, 2146), β-actin (CST, 4970), Gapdh (CST, 2118), iNOS (CST, 13120), Il1β (CST, 12242), Cd63 (Abcam, ab217345), Tsg101 (Abcam, ab30871), Calnexin (Abcam, ab22595), Hsp70 (Abcam, ab181606), Hif1α (Novus, NBP1-02160), Cxcr4 (Abcam, ab124824), Vegf (Abcam, ab46154), Socs1 (CST, 3950), NFκB p65 (CST, 8242), p-NFκB p65 (CST, 3033), and Irf1 (CST, 8478). Proteins were visualized with horseradish peroxidase (HRP)-conjugated anti-rabbit, anti-mouse IgG (CST), followed by the use of the ECL chemiluminescence system.

### Flow cytometry

To analyze the frequencies of M1-activated microglia (CD11b^+^ CD86^+^), mouse primary microglia or BV2 cells were stained with fluorochrome-labeled antibodies specific for CD11b (FITC, #101205) and CD86 (PE, #105007). The cells were stained with surface markers for 30 minutes at 4 °C and analyzed by flow cytometry (BD LSRFortessa). The results were assessed using FlowJo (version 10.0.7, Tree Star, Ashland, OR, USA).

### Cell viability assay (CCK8)

The viability of HUVECs treated with exosomes or PBS was assessed by a CCK8 assay (Dojindo, Japan). The CCK8 reagent was applied to each well, and the cells were cultured at 37 °C for 3 hours. At 450 nm, the absorbance (optical density) was measured. The optical density ratio of a treated culture over an untreated control was used to calculate cell viability, which was then quantile normalized in the log2 scale.

### Wound healing and tube formation assay

The rate of HUVECs migration was measured using wound healing assays. A total of 4 × 10^4^ HUVECs were seeded in a 12-well plate and treated with PBS or exosomes for 48 hours. The cell monolayer was then manually scraped with a 200-μL pipet tip to create the wound. Images taken at 0 hours following wounding, when the wound size had stabilized, were used to quantify the initial wound. Additional photos were taken at random wound sites 24 and 48 hours after wounding. Using Image-Pro Plus software, each sample was quantitatively evaluated. The cell migration distance was calculated by comparing the wound area under various experimental circumstances to the wound area under control conditions. For the tube formation assay, the Matrigel matrix (Corning) was plated in a 96-well plate and incubated at 37 °C for 30 minutes to allow the Matrigel to polymerize. A total of 4 × 10^4^ HUVECs treated with exosomes or PBS were seeded on the Matrigel-coated well. The plate was incubated in a humid environment containing 5% CO_2_ at 37 °C. Tube formation was observed at 12 hours with the microscope. The number of tubes formed was used to measure tube forming ability.

### The enzyme-linked immunosorbent assay (ELISA)

The cells were cultured for 48 hours in fresh media without serum. The supernatant was collected and centrifuged for 10 minutes at 1000 rpm. The supernatant was aliquoted into microcentrifuge tubes after centrifugation for additional mouse CXCL12/SDF-1 ELISA (Elabscience, China).

### Experimental animal design

C57BL/6J mice (half male and half female) at the age of 6-8 weeks were randomly divided into four groups: negative control (PBS), operation control (sham), M0-EXO intravitreal injection (M0-EXO), and M1-EXO intravitreal injection (M1-EXO). PBS was used as a negative control, and the sham-operated group was established to eliminate the impact of the invasive operation itself. The mice were then anesthetized, and a 2.5-μL 34G Hamilton syringe (Hamilton, Reno, NV, USA) was used to make intravitreal injections; specifically, 1 μL of microglial exosome solution (1 mg/mL) was injected into the vitreous cavity of the right eye of M0-EXO or M1-EXO group, and 1 μL of PBS was injected into the right eye of PBS group. The intravitreal operation without injection was performed on the sham group. The injection operations were performed every three days for six weeks. The minocycline treatment was initiated one day prior to the intravitreal injection (45 mg/kg per day dissolved in PBS) 28.

### Retinal flat-mount staining

The cornea and lens were removed following enucleation, and the eyes were then fixed in 4% PFA for an entire night. The retina was then dissected by separating the choroid, sclera, and vitreous layers. The retina was stained with Iba1 (Abcam, ab178847) antibody overnight at 4 °C and then treated for 1.5 hours at room temperature with Alexa Fluor 488-conjugated anti-rabbit antibody (CST, 4412, 1:500). The retina was flattened and placed onto slides by making radial incisions. A fluorescent microscope was used to image flat mounts at 20×magnification (Axio Imager Z1, Carl Zeiss, Germany).

### Retinal cryosection staining and immunofluorescence assay

After the anterior segments were removed, the posterior eyecup was fixed in 4% PFA overnight and treated in 10% sucrose for two hours before being incubated in 30% sucrose overnight. Eyecups were imbedded in optimal cutting temperature compound (OCT, Tissue Tek, USA) and cryosections were cut at 10 μm intervals. Cells grown on glass coverslips or tissue slices were fixed for 10 minutes at room temperature in 4% paraformaldehyde. PBS containing 1% bovine serum albumin and 0.2% Triton X-100 were added for 30 minutes. The following primary antibodies were used: Pecam1 (Santa Cruz, sc-46694), Iba1 (Abcam, ab178847), Sdf1 (Abcam, ab25117), Cxcr4 (Abcam, ab181020), Ki67 (CST, 9129). Then, the samples were stained with secondary antibodies for 1 hours at 37 °C).

### Real-time reverse transcription-polymerase chain reaction (RT-PCR) analysis

TRIzol Reagent was used to isolate total RNA from cells or tissues (Invitrogen, Carlsbad, CA). Exosome total RNA was extracted using the TRIzol Reagent (Invitrogen, Carlsbad, CA) and Dr. GenTLETM Precipitation Carrier (Takara, China). Reverse transcription-polymerase chain reaction (RT-PCR) assays were performed according to the manufacturer's protocol for the SYBR Prime Script TM RT-PCR Kit or Mir-X™ miRNA First-Strand Synthesis Kit (Takara, China). The Roche 480 system was used for real-time PCR (Roche, USA). The 2^-ΔΔCt^ method was used for the relative quantification of target gene expression levels. The primers of RNA are shown in Table S1.

### si-RNA, miRNA agomiR/antagomir, and Plasmid Transfection

*In vitro*, miR-155-5p agomiR/ antagomiR, mall interfering RNA (siRNA) against Socs1 (si-Socs1), and nonspecific control siRNA (si-NC) were obtained from RiboBio (Guangzhou, China) and performed according to the manufacturer's protocol. The BV2 cells were transfected with plasmid pEnCMV-MCS (empty vector, cat. no. p8196, Miaolingbio, China) or plasmid pEnCMV-Irf1 (Irf1 overexpression, P30418, Miaolingbio, China) by Lipofectamine® 3000 Transfection Reagent (Invitrogen, Carlsbad, CA, USA) according to the manufacturer's protocol.

### miRNA-targets prediction

miRNAs obtained from previous experimental results were selected. To improve the accuracy of prediction results, five popular databases, TargetScan, miRanda, miRDB, PITA, and PicTar, were used to perform intersection. The target genes identified by all databases were chosen for further investigation.

### Preparation of miRNA mimic/inhibitor-loaded exosomes

According to a previous study, a modified method of calcium chloride transfection was used 29. 200 pmol miRNA mimic or inhibitor was mixed with 20 μg exosomes in PBS for a 60-mm plate (0% confluence) with 5 mL of exosome-free medium, then CaCl2 (final concentration 0.1 M) was added. Using sterile PBS, the final volume was adjusted to 300 μL. The mixture was placed on ice for thirty minutes. After being heat-shocked at 42 °C for 1 minute, the mixture was placed on ice for 5 minutes. For the RNase treatment, exosomes are incubated with 5 μg/mL of RNase for 30 minutes at 37°C.

### Luciferase Reporter Assay

Dual-Luciferase Reporter Assay System (Promega, Madison, WI, USA) was used to measure luciferase assays 48 hours after transfection. The activity of Firefly luciferase was adjusted to that of Renilla luciferase.

### Exosomal miRNA and cellular mRNA sequencing

The exosomal miRNA-Seq and cellular mRNA-Seq experiments were performed by Epibiotek company (Guangzhou, China). According to the manufacturer's manual, approximately 50 ng of total exosomal RNA was used to construct a library using the NEBNext Multiplex Small RNA Library Prep Set for Illumina (Illumina, San Diego, CA). Libraries were then amplified and sequenced using HiSeq Rapid SBS Kit V2 (50 cycles) and HiSeq Rapid SR Cluster Kit V2 at the HiSeqTM 2500 system (Illumina).

### Statistical analysis

All *in vitro* experiments were conducted at least three times. The number of mice in the *in vivo* study was at least six in each group. The data were shown as the means ± SDs. R program (version 4.0.3) was used to calculate statistical significance. We utilized the Shapiro-Wilk and Levene tests to assess the normality and homogeneity of variance. The Student's two-tailed t-test (for two groups) or analysis of variance (ANOVA, for more than two groups) was used to assess differences in mean values. A *P*<0.05 (two-sided) difference was deemed to be statistically significant.

## Results

### Intercellular communication from M1 microglia toward M0 microglia was increased through exosomes in the OIR model by bioinformatics analysis

**Figure 1:**
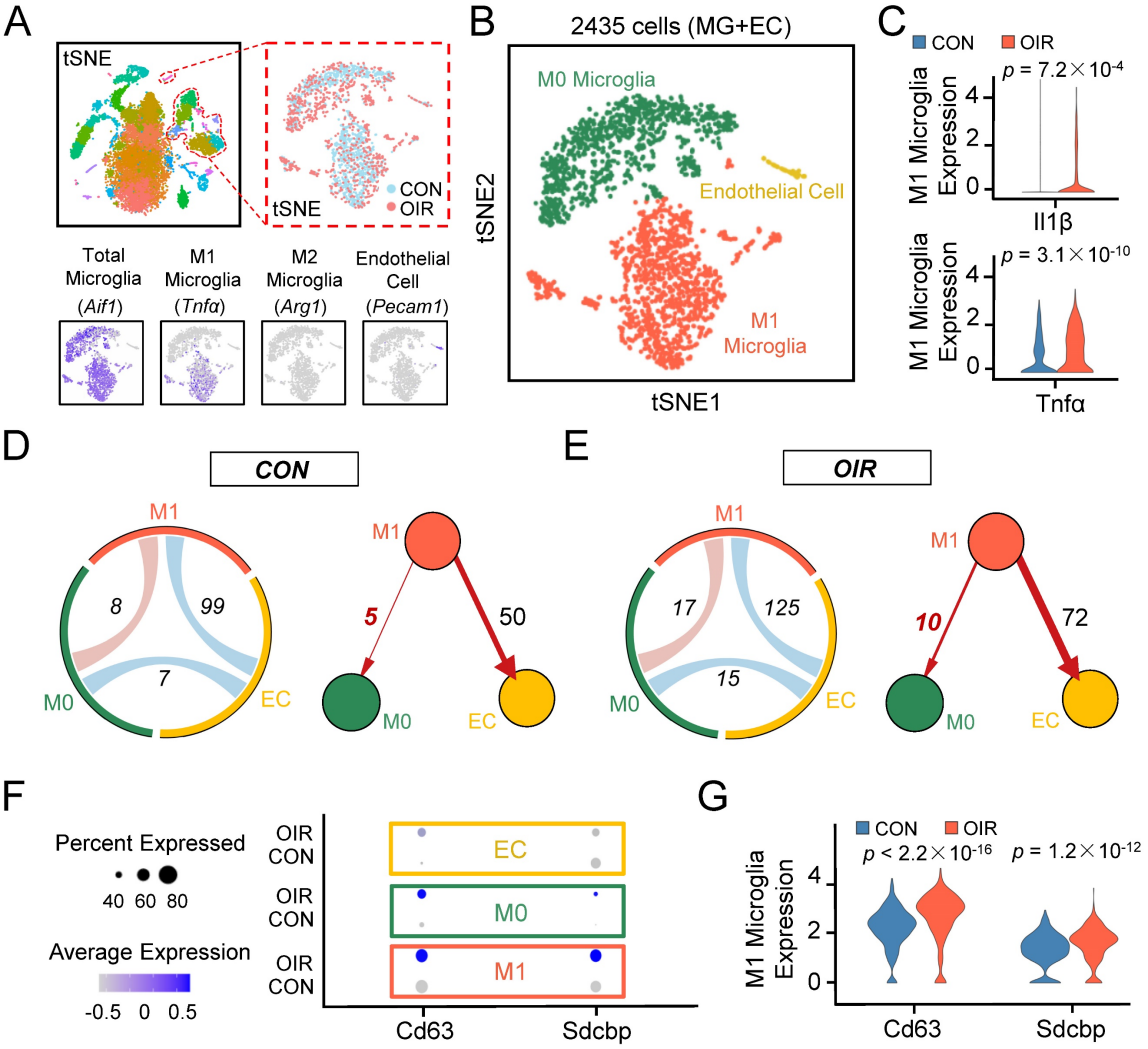
** Single-cell RNA-seq uncovers that intercellular communication from M1 microglia toward M0 microglia is increased in the retinal angiogenesis model.** (A) t-SNE plots of the total 15185 single cells. Lower panels: feature plots show expression of some of the genes used to define clusters. (B) t-SNE plots of 2435 cells (endothelial cells plus various types of microglia) in normal and OIR. (C) The expression of M1 activated microglial markers like Il1β and Tnfα were increased in M1 microglia from OIR. (D and E) The chord diagram and directed graph show that cell-to-cell communications among various cells were elevated, and intercellular communications from M1 microglia toward ECs or M0 microglia increased in OIR. The orange-red represents M1 microglia, the green represents M0 microglia, and the yellow represents ECs. (F) Feature expression heatmap of two known marker genes for exosomes across the identified three cell clusters. (G) The expression of two known marker genes for exosomes were increased in M1 microglia from OIR. *P* values were determined by the Mann-Whitney U test with false discovery rate correction. t-SNE: t-distributed scholastic neighbor embedding; CON: control; OIR: oxygen-induced retinopathy; ECs: endothelial cells.

The OIR model allows the angiogenic response of the retina to be directly interrogated 12. To better understand the potential contribution of microglia to retinal angiogenesis, we analyzed the transcriptomes of single cells extracted from the retinae of control and OIR mice from postnatal Day 17 based on a publicly available single-cell RNA sequencing dataset (accession no. GSE152928). After quality control filters, 9045 and 6140 retinal cells were obtained from control and OIR mice, respectively. We performed unbiased clustering of cell profiles and found 36 clusters **(Figure S1A)**. According to multiple well-established markers, we found that microglia/macrophage-related genes were primarily distributed in eight clusters, and a cell cluster exhibited the characteristics of endothelial cells (ECs). With known microglia subtype-specific markers, we further grouped microglia/macrophage-related clusters into two major classes, including M0 microglial cluster and M1 microglial cluster **(Figure 1A-B, Figure S1A-C)**. Il1β and Tnfα, two characterized genes of proinflammatory M1 polarization, were increased explicitly in the M1 microglial cluster in the OIR retina (**Figure 1C**, Il1β: ***P<0.001; Tnfα: ***P<0.001), suggesting that the M1 microglial cluster had a higher level of polarization and a more active state in pathological processes. To further account for the effects of active M1 microglia on various cells, we mapped receptor‒ligand pairs onto cell types to construct a putative cell‒cell communication. We observed that M1 microglial activity was enhanced in the retinal cell‒cell communication networks of OIR mice compared to control mice (M1-EC: CON: 99; OIR: 125; M1-M0: CON: 8; OIR: 17) **(Figure 1D-E)**. Specifically, intercellular communication from M1 microglia toward vascular ECs (M1 to ECs: CON: 50; OIR: 72) or M0 microglia (M1 to M0: CON: 5; OIR: 10) was increased. Because many studies have reported connections between activated microglia and ECs in angiogenesis, we focused on the relationship between M1-polarized microglia and microglia in a resting homeostatic state 30,31. However, the communication mechanism between activated and resting microglia remains unclear.

Previous studies have demonstrated that exosomes are important mediators of cell‒cell communication, and exosome internalization can be mediated by ligand‒receptor-mediated endocytosis 32-35. Therefore, exosomes derived from M1 microglia could be essential in resting microglia in OIR. Consistent with our expectations, the specific exosomal markers Cd63 36 and Sdcbp 37 were significantly increased in the M1 microglial cluster from the OIR retina (Cd63: ***P<0.001; Sdcbp: ***P<0.001) **(Figure 1F-G)**. Thus, these findings suggest that intercellular communication from M1 microglia toward M0 microglia was increased in OIR-induced angiogenesis, and M1 microglia-derived exosomes probably mediated the process above.

### M1 microglial exosomes promote microglia M1 polarization *in vitro*

**Figure 2:**
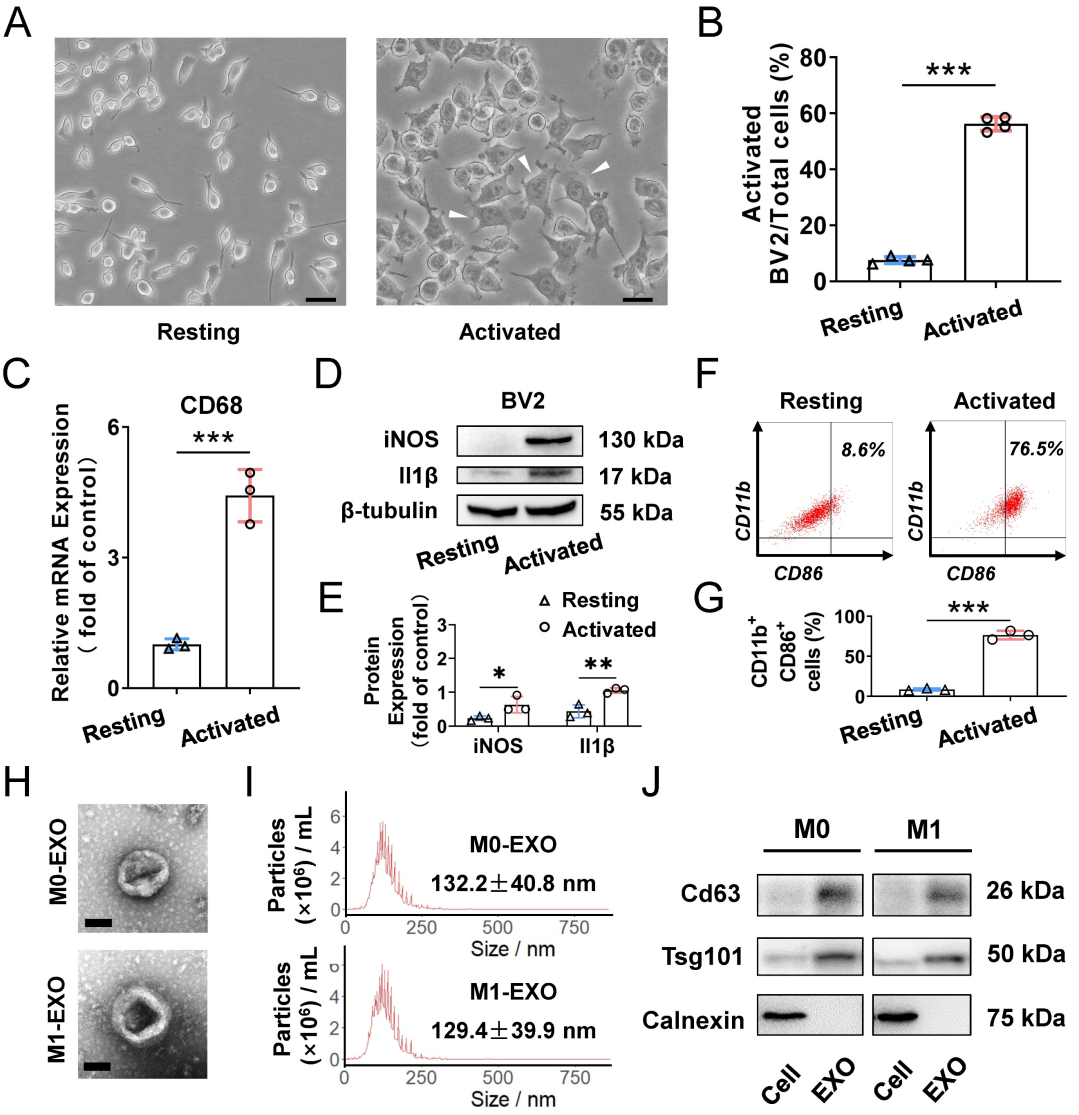
** Characterization of exosomes derived from resting and LPS-activated M1 microglia.** (A) Representative microscopy images of resting and activated microglia, and many multipolar cells which have a large soma (white arrowheads) appear after LPS treatment. Scale bar, 30 μm. (B) The percentages of activated microglia per field with multipolar and ameboid shapes were quantified (n ≥ 3, ***P<0.001). (C) The mRNA levels of CD68 in LPS treatment and negative control groups were determined by qRT-PCR (n = 3, ***P<0.001). (D) Western blot images showing the protein levels of iNOS and Il1β were increased in M1 microglia compared to M0 microglia. (E) Relative quantification of protein expression in microglia (n = 3, **P<0.01, *P<0.05). (F) LPS increased M1 phenotype (CD11b- and CD86-positive) switching in BV2 microglia. (G) Statistical analysis of the percentage of CD11b- and CD86-positive cells of LPS-stimulated BV2 microglia cells (n = 3, ***P<0.001). (H) Representative transmission electron microscopy images of M0 and M1 microglia-derived exosomes. Scale bar, 100 nm. (I) Nanoparticle tracking analysis indicated the average size of M0 and M1 microglia-derived exosomes. (J) Western blot analysis of the different protein markers (Cd63, Tsg101) of exosomes collected from various types of microglia. Calnexin was used as an negative control. EXO: exosomes; qRT-PCR: quantitative real-time reverse transcription-polymerase chain reaction.

To clarify the role of M1-polarized microglia-derived exosomes, we utilized BV2 cells, a type of mouse microglial cell, for further experiments. BV2 cells were polarized to the M1 phenotype by adding 100 ng/ml LPS, which is a typical method to activate microglia 38. As shown in **Figure 2A**, LPS treatment promoted cells to change to an irregular form with numerous pseudopodia. The proportion of activated microglial cells was increased compared with PBS addition in the control groups (Resting: 7.4±1.1%; Activated: 53.0±4.0%, ***P<0.001)** (Figure 2B)**. Moreover, the expression of activated microglia markers, including CD68 for microglia activation, as well as iNOS and Il1β for M1 phenotype, were all significantly increased (CD68: Resting: 1.005±0.128; Activated: 4.422±0.601, ***P<0.001; iNOS: Resting: 0.234±0.067; Activated: 0.636±0.240, *P<0.05; Il1β: Resting: 0.440±0.191; Activated: 1.055±0.077, **P<0.01)** (Figure 2C-E)**. Furthermore, based on analysis of CD11b- and CD86-positive (another marker expressed by M1 phenotype microglia) cells by using flow cytometry, we found that LPS significantly induced the BV2 microglia to polarize to a M1 phenotype (Resting: 8.6±1.3%; Activated: 76.5±5.4%, ***P<0.001) **(Figure 2F-G)**. Therefore, the M1 activation pattern of BV2 cells treated with LPS is consistent with that reported in a previous study 39.

To better understand the functions of exosomes derived from M0- and M1-type BV2 cells, we first isolated exosomes by ultracentrifugation for identification. The typical cup-shaped membrane vesicle morphology was observed under transmission electron microscopy (TEM) **(Figure 2H)**. Nanoparticle tracking analysis (NTA) indicated that the size distribution of exosomes derived from M0 and M1 BV2 cells mainly ranged from 30 to 150 nm (M0-EXO: 132.2±40.8 nm; M1-EXO: 129.4±39.9 nm) **(Figure 2I)**. Western blot profile showed that known exosome characteristic markers, including Cd63 and Tsg101, were highly abundant in exosomes. Moreover, the cell lysate was enriched with Calnexin, an endoplasmic reticulum protein 40,41, whereas Calnexin was not detected in exosomes, indicating that the isolated exosomes were relatively pure without contamination of other cell compartments **(Figure 2J)**.

**Figure 3:**
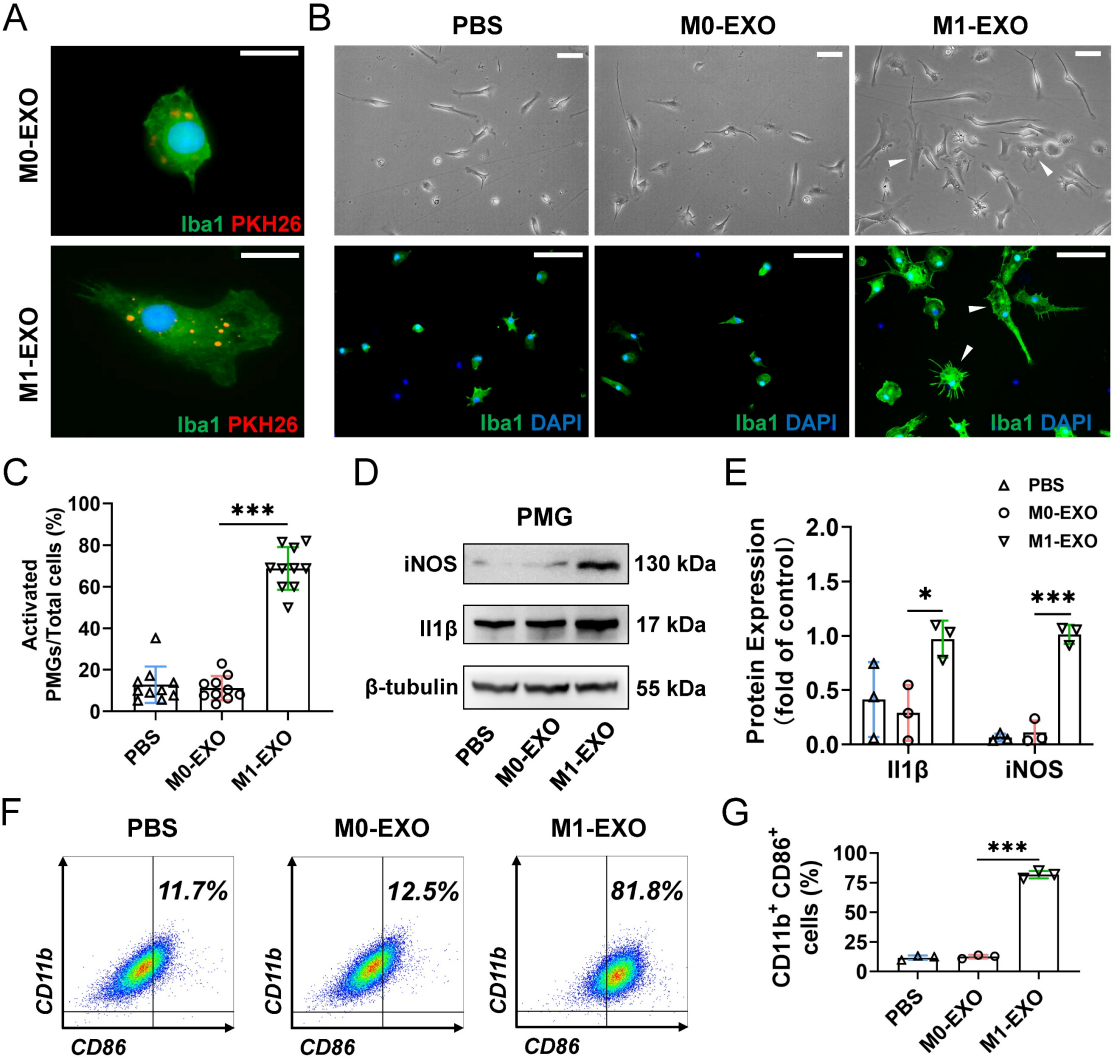
** M1 microglial exosomes promote the M1 polarization of primary retinal microglia* in vitro*.** (A) Exosomes labeled with PKH26 were engulfed by PMGs (green, Iba1; red, PKH26). Scale bar, 10 μm. (B) Morphology changes of PMGs following treatment with M0-EXO or M1-EXO. Upper panel: microscopy images; lower panel: immunofluorescent images. White arrowheads: activated microglia. Scale bar, 20 μm. (C) The percentages of activated PMGs per field with multipolar and ameboid shapes were quantified (n ≥ 6, ***P<0.001). (D) Western blot images show that the protein levels of iNOS and Il1β were increased after M1-EXO treatment. (E) Relative quantification of protein expression in PMGs (n = 3, ***P<0.001, *P<0.05). (F) Percentage of CD11b^+^ CD86^+^ PMGs among the three groups. (I) Statistical analysis of the percentage of CD11b- and CD86-positive cells of PMGs (n = 3, ***P<0.001). PMGs: primary microglia; EXO: exosomes.

We next focused on the effect of exosomes on primary retinal microglial cells (PMGs). Cultured PMGs were incubated with PKH26-labeled exosomes (red), which were collected from the BV2-derived conditioned medium. At 24 hours after treatment, the cells were stained with Iba1^+^ (a specific microglial marker). Our data showed that both M0-EXO and M1-EXO (red) were localized in the cytoplasm of Iba1-positive PMGs (green), suggesting that exosomes were engulfed by PMGs **(Figure 3A)**. Moreover, treatment with M1-EXO in PMGs for 24 hours induced cellular morphological changes (white arrowheads) **(Figure 3B)**. Compared with the PBS group and M0-EXO-treated group, more amoeboid and irregularly shaped Iba1-postive cells were observed in the group treated with M1-EXO (PBS: 0.129±0.088; M0-EXO: 0.112±0.058; M1-EXO: 0.688±0.103 *F_2,27_*=149.2, ***P<0.001) **(Figure 3C)**. To characterize the polarized phenotype of activated microglia, we investigated the expression of mRNA for different activation markers. As shown in **Figure S2A-B**, M1-EXO treatment could promote microglia M1 phenotype, rather than M2 phenotype. We further measured the expression levels of the M1-activated markers iNOS and Il1β by Western blotting. The results showed that compared with the PBS group and M0-EXO-treated group, the expression of iNOS and Il1β was significantly increased in the M1-EXO group (iNOS: PBS: 0.064±0.035; M0-EXO: 0.112±0.111; M1-EXO: 1.014±0.087, *F_2,6_*=121.0, ***P<0.001; Il1β: PBS: 0.414±0.345; M0-EXO: 0.292±0.256; M1-EXO: 0.970±0.170, *F_2,6_*=5.516, *P<0.05) **(Figure 3D-E)**. In addition, as presented in **Figure 3F-G**, the results of flow cytometry further confirmed that M1-EXO treatment significantly increased in the percentage of M1 microglia (PBS: 11.7±1.9%; M0-EXO: 12.5±1.5%; M1-EXO: 81.8±3.2%, *F_2,6_*=935.6, ***P<0.001). Taken together, our results suggest that PMGs were polarized toward a M1 proinflammatory state after M1 exosomal treatment* in vitro*.

### M1 microglia enhanced the proangiogenic potential of M0 microglia through exosomes *in vitro*

**Figure 4:**
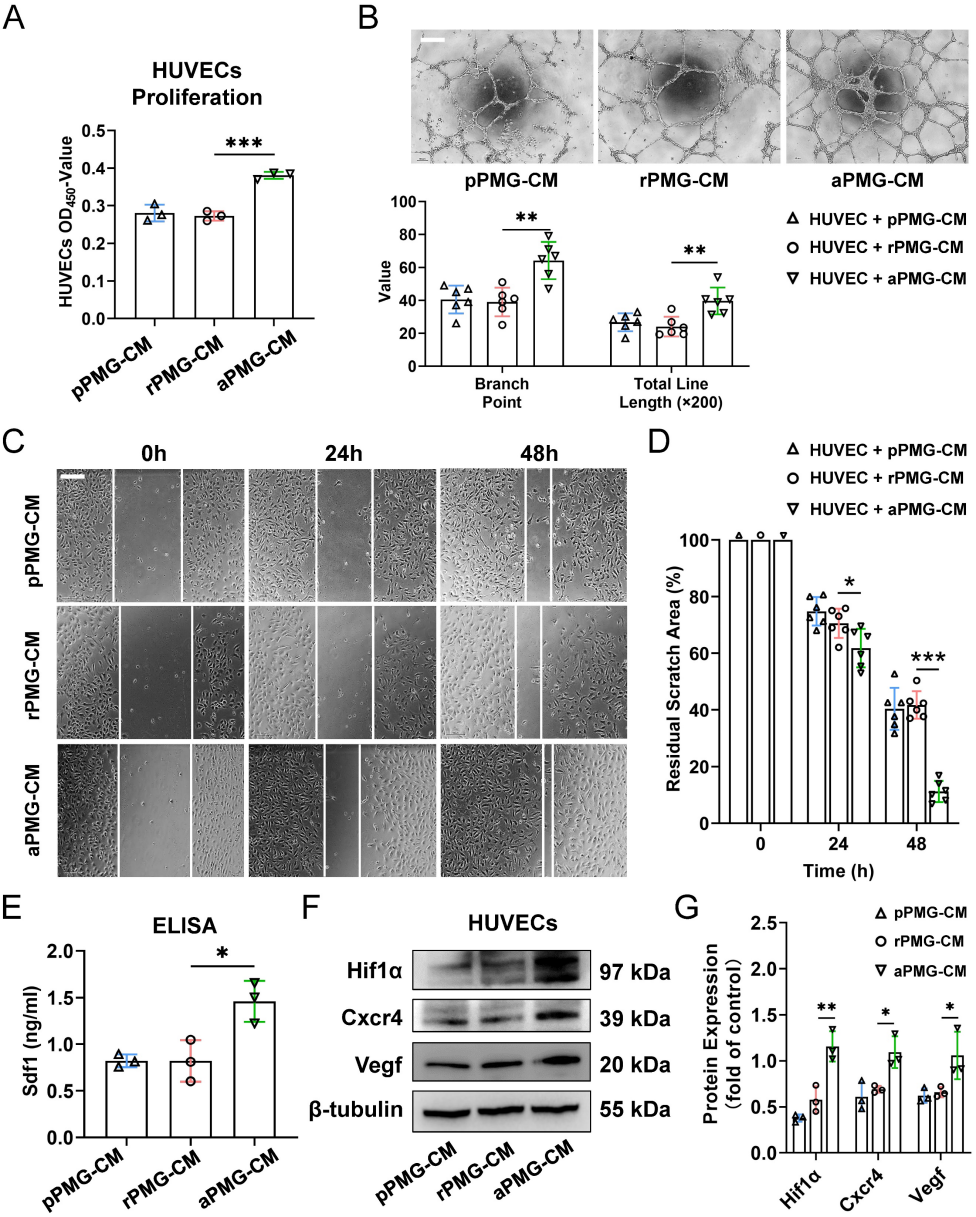
** Exosomes derived from M1 microglia elevate the proangiogenic ability of microglia *in vitro.*** (A) HUVECs proliferation was assessed using CCK8 assays after pPMG-CM, rPMG-CM, or aPMG-CM treatment (n = 3, ***P<0.001). (B) Upper panel: representative microscopic images of tube formation on Matrigel; lower panel: the quantification of branch points and total line length (×200). There was a significant increase in HUVECs tubule connections in the presence of aPMG-CM compared with that in the presence of pPMG-CM or rPMG-CM (n = 6, **P<0.01). (C) Wound-healing assay. HUVECs were treated with pPMG-CM, rPMG-CM, or aPMG-CM, and cell monolayers were scratched with 200 μl yellow tips. Images were captured at 0, 24, and 48 h after the wound scratch. (D) The ratio of the residual scratch area was measured by comparing the area at each time point with the initial area (n = 6, ***P<0.001, *P<0.05). (E) ELISA results show that the concentration of Sdf1 was increased after aPMG-CM stimulation compared with the other groups (n = 3, *P<0.05). (F)Western blot images showing the protein levels of Hif1α, Cxcr4, and Vegf were increased in HUVECs after aPMG-CM stimulation compared with the other groups. (G) Relative quantification of protein expression in HUVECs (n = 3, **P<0.01, *P<0.05). HUVECs: human umbilical vein endothelial cells; PMGs: primary microglia; pPMG-CM: PBS-treated PMG-conditioned medium; rPMG-CM: resting exosomes-treated PMG-conditioned medium; aPMG-CM: activated exosomes-treated PMG-conditioned medium.

To assess the proangiogenic ability of PMGs after treatment with microglial exosomes, HUVECs were treated with the conditioned medium described below. For example, PMGs were treated with M0-EXO, M1-EXO, and PBS as controls. Then, the conditioned medium was collected as PBS-treated PMG-conditioned medium (pPMG-CM), resting exosome-treated PMG-conditioned medium (rPMG-CM), and activated exosome-treated PMG-conditioned medium (aPMG-CM). As shown in **Figure 4A**, aPMG-CM significantly promoted the proliferation of HUVECs compared to cells treated with pPMG-CM or rPMG-CM (pPMG-CM: 0.281±0.022; rPMG-CM: 0.273±0.013; aPMG-CM: 0.381±0.009, *F_2,6_*=43.87, ***P<0.001). Similarly, compared with pPMG-CM or rPMG-CM treatment, aPMG-CM treatment increased the number of capillary-like tubes and junctions. After quantification, rPMG-CM significantly strengthened tube formation (branch point: pPMG-CM: 40.500±8.479; rPMG-CM: 39.000±8.672; aPMG-CM: 64.167±11.286, *F_2,15_*=13.07, ***P<0.001; total line length: pPMG-CM: 26.662±5.488; rPMG-CM: 24.078±5.995; aPMG-CM: 39.622±8.178, *F_2,15_*=9.393, **P<0.01) **(Figure 4B)**. The effect of CM on HUVECs migration was evaluated by a migration wound-healing assay. The results showed that a significantly smaller remaining wound area was observed under light microscopy at 24 hours and 48 hours after treatment with aPMG-CM (24 hours: pPMG-CM: 74.787±5.037; rPMG-CM: 70.540±5.178; aPMG-CM: 61.789±6.817, *F_2,15_*=8.015, **P<0.01; 48 hours: pPMG-CM: 40.351±7.409; rPMG-CM: 41.697±4.872; aPMG-CM: 11.223±3.750, *F_2,15_*=57.58, ***P<0.001) **(Figure 4C-D)**.

Moreover, the whole protein was extracted from HUVECs 24 hours after treatment to evaluate the expression of angiogenesis-related proteins. The ELISA showed that the expression of Sdf1 was significantly increased in the aPMG-CM group compared with the pPMG-CM or rPMG-CM groups (pPMG-CM: 0.823±0.068; rPMG-CM: 0.821±0.223; aPMG-CM: 1.460±0.220, *F_2,6_*=11.94, **P<0.01) **(Figure 4E)**. Moreover, as shown in Figure **4F-G**, Western blot assays indicated that Cxcr4, Hif1α, and Vegf were all significantly increased in HUVECs treated with aPMG-CM (Hif1α: pPMG-CM: 0.378±0.039; rPMG-CM: 0.579±0.144; aPMG-CM: 1.158±0.165, *F_2,6_*=29.64, ***P<0.001; Cxcr4: pPMG-CM: 0.611±0.158; rPMG-CM: 0.691±0.037; aPMG-CM: 1.095±0.174, *F_2,6_*=10.71, *P<0.05; Vegf: pPMG-CM: 0.623±0.077; rPMG-CM: 0.660±0.056; aPMG-CM: 1.058±0.258, *F_2,6_*=6.937, *P<0.05).

Collectively, these data demonstrated that M1-EXO-treated microglia promoted angiogenesis *in vitro*; in other words, M1 microglial exosomes activated M0 microglia, subsequently amplified its proangiogenic activity, which suggests an indirect link between angiogenesis and exosomes derived from activated microglia.

### M1 microglial exosomes promote the polarization of M1 retinal microglia *in vivo*

**Figure 5:**
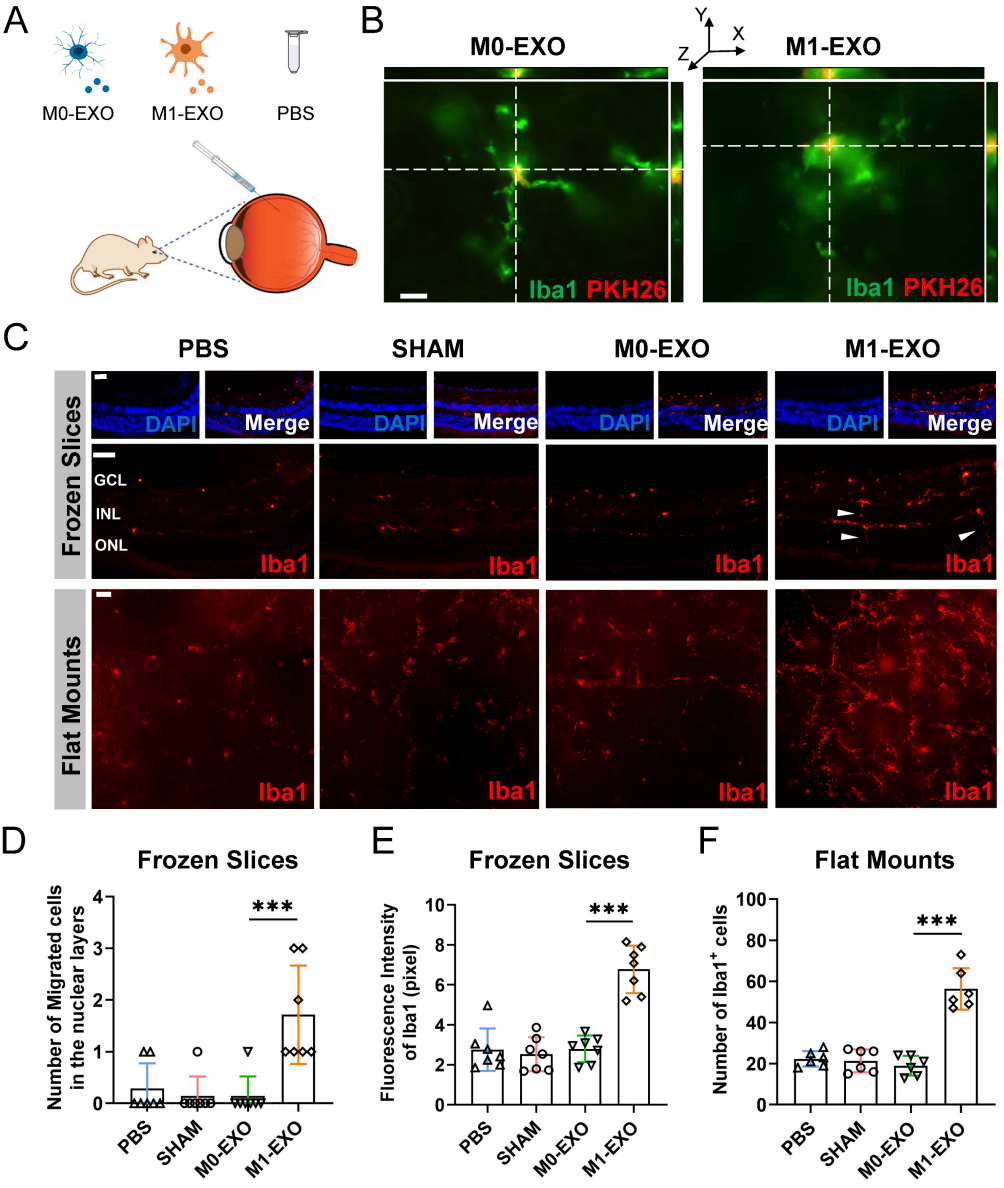
**M1 microglial exosomes promote retinal microglia activation *in vivo*.** (A) Schematic diagram of the adult mice models with the treatment of PBS, M0-EXO, and M1-EXO. (B) Representative 3D confocal images show that PMGs swallowed exosomes (M0-EXO or M1-EXO) labeled with PKH26 (green, Iba1; red, PKH26). Scale bar, 20 μm. (C) Representative images of immunofluorescence staining (upper panel) and flat mount (lower panel) stained with Iba1 (red) in different treatment groups. White arrowheads: activated and migrated microglia. Scale bar, 50 μm. Quantification of the number of migrated microglia (D), fluorescence intensity (E) based on frozen slices, and quantification of the number of Iba1^+^ cells based on flat mount in various groups are shown (n ≥ 6, ***P<0.001, *P<0.05). EXO: exosomes; PMGs, primary microglia.

To confirm the results *in vitro*, we examined the changes of the retina after the intravitreal injection of M0-EXO or M1-EXO with the negative controls of PBS and sham group, using adult mouse model (Figure 5A). As shown in Figure 5B, PKH26-labeled exosomes (red) were detected by 3D confocal images in the mouse retina two days after intravitreal injection, and exosomes were localized to the cytoplasm of Iba1+ microglia (green). At six weeks after injection, the retinas were frozen and cut into slices or radially cut and mounted onto slides and then stained with an antibody against Iba1-postive (red) (Figure 5C). We found that the number of migrated microglia was increased after treatment with M1-EXO in the nuclear layer (PBS: 0.286±0.488, SHAM: 0.143±0.378, M0-EXO: 0.143±0.378, M1-EXO: 1.714±0.951, F3,24=11.47, ***P<0.001) (Figure 5D), which is a typical cell feature of activated microglia 27. Moreover, the quantification of cell density showed that M1-EXO treatment dramatically increased the polarized level of microglia, whereas there was no significant difference among the other groups (frozen slices: PBS: 2.755±1.059, SHAM: 2.527±0.848, M0-EXO: 2.792±0.667, M1-EXO: 6.774±1.187, *F_3,24_*=31.69, ***P<0.001; flat mounts: PBS: 22.33±3.777, SHAM: 21.33±5.574, M0-EXO: 19.00±4.817, M1-EXO: 56.33±10.11, *F_3,20_*=44.41, ***P<0.001) (Figure 5E-F). These findings demonstrate that exosomes derived from M1 BV2 cells promote the activation of retinal resident microglia, which is consistent with the *in vitro* results.

### M1 microglial exosomes induce angiogenesis following activation of retinal microglia *in vivo*

**Figure 6:**
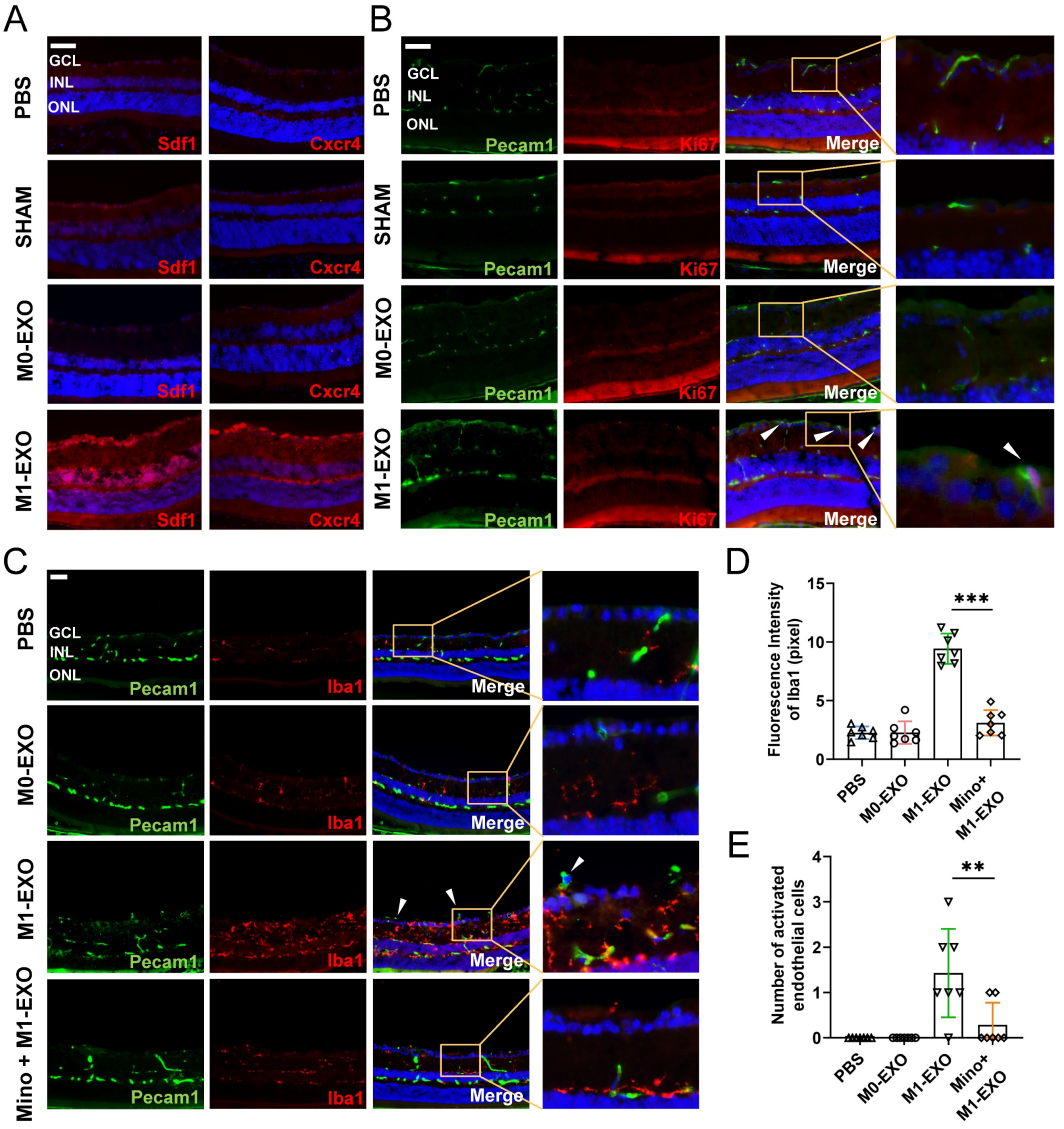
** Exosomes derived from M1 microglia promote angiogenesis *in vivo*, and suppressing the activation of retinal microglia reverses the effects.** (A) Representative images of immunofluorescence staining for DAPI (blue), Sdf1 (red, left panel) and Cxcr4 (red, right panel). Sdf1 and Cxcr4 were enormously increased after the intravitreal injection of M1-EXO compared to the other groups. Scale bar, 50 μm. (B) Representative images of double-staining for Pecam1 (green) and Ki67 (red) in PBS-injected, SHAM, M0-EXO, or M1-EXO groups. Scale bar, 50 μm. (C) Double-staining for Pecam1 (green) and Iba1 (red) in PBS-injected, M0-EXO, M1-EXO, or Minocycline plus M1-EXO groups. Minocycline treatment before M1-EXO administration effectively suppressed M1-EXO induced microglia activation and abnormal EC proliferation (white arrowheads). Bar = 50 μm. (D) Quantification of fluorescence intensity showed that M1-EXO induced microglia activation, and Minocycline pretreatment alleviated this upregulation (n ≥ 6, ***P<0.001). (E) Quantification of the number of abnormal EC in various groups (n ≥ 6, **P<0.01). EXO: exosomes; GCL: ganglion cell layer; INL: inner nuclear layer; ONL: outer nuclear layer; EC: endothelial cell; Mino: minocycline.

Microscopic clues about retinal neovascularization occurred after M1-EXO intravitreal injection in adult mouse model. Immunofluorescence analysis showed that both Cxcr4 and Sdf1, markers of angiogenesis 42, showed faint nonspecific staining along the outer border of the retina and in the choroid after PBS, SHAM, or M0-EXO treatment. In contrast, Cxcr4 and Sdf1 were strongly positive in sections from M1-EXO-injected mice (Figure 6A). Moreover, retinal vascular endothelial cell proliferation is a biological process involved in pathological neovascularization 43. Therefore, the retinal tissue sections were further double-stained with antibodies against Pecam1 and Ki67, the former being a marker for vascular endothelial cells and the latter for proliferating cells. After counting Ki67- and Pecam1-positive cells that extended anteriorly to the inner limiting membrane per section, endothelial cell proliferation was only observed in the M1-EXO treatment group compared with the other three groups (Figure 6B). Consistent with the* in vitro* results, M1 microglial exosomes promoted retinal abnormal endothelial cell proliferation *in vivo.*

Considering the complexity of the retinal microenvironment *in vivo*, to further demonstrate the hypothesis that M1-EXO might activate resting microglia thus exaggerating microglia-mediated angiogenesis, we used minocycline, a typical microglial activation inhibitor, to establish an intervention group 44,45. As shown in Figure 6C, minocycline treatment significantly decreased the number of activated microglia (PBS: 2.265±0.537, M0-EXO: 2.275±0.958, M1-EXO: 9.432±1.288, Mino+M1-EXO: 3.116±1.085, *F_3,24_*=83.05, ***P<0.001) (Figure 6D). Moreover, minocycline significantly inhibited Pecam1+ cells from breaking through the internal limiting membrane induced by M1-EXO (PBS: 0.000±0.000, M0-EXO: 0.000±0.000, M1-EXO: 1.429±0.976, Mino+M1-EXO: 0.286±0.488, *F_3,24_*=10.88, ***P<0.001) (Figure 6E). These results suggest that minocycline alleviated the proangiogenic effect of M1-EXO by suppressing the activation of retinal microglia and provide substantial evidence to suggest that the proangiogenic effect of M1 microglial exosomes is mediated by activated retinal microglia. In other words, M1-EXO amplifies microglia-related proangiogenic effects by activating M0 microglia. However, the precise mechanisms remain to be elucidated.

### M1 microglial exosomes induce resting microglial activation via exosomal miR-155-5p

**Figure 7:**
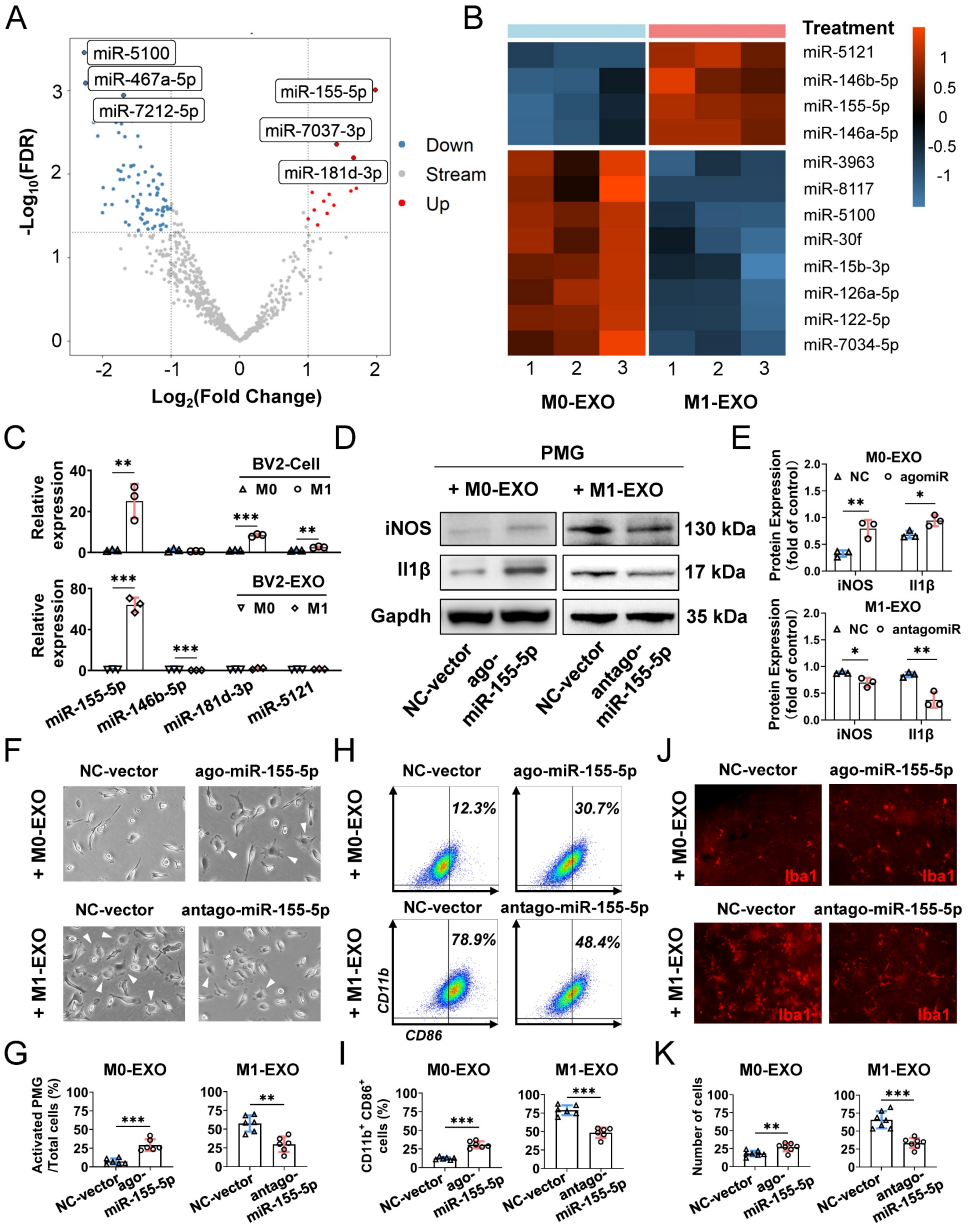
** Exosomal miR-155-5p from M1 microglia induced resting microglia activation both *in vitro* and *in vivo*.** (A) The volcano plot shows the variation in miRNA expression between M0-EXO and M1-EXO. Red in the heatmap denotes upregulation; blue denotes downregulation. (B) The heatmap shows differential exosomal miRNA between resting and activated microglia after miRNA sequencing. (C) The RNA levels of miR-155-5p, miR-146b-5p, miR-181d-3p, and miR-5121 in BV2 microglia and exosomes before and after activation were determined by qRT-PCR (n = 3, ***P<0.001, **P<0.01). (D) Western blot images show the protein levels of iNOS and Il1β in PMGs after treatment with M0-EXO, agomir-155-5p transfected M0-EXO, M1-EXO, and antagomir-155-5p transfected M1-EXO. (E) Relative quantification of protein expression in PMGs (n = 3, **P<0.01, *P<0.05). (F) Representative microscopy images show the morphology changes of PMGs following treatment with M0-EXO, agomir-155-5p transfected M0-EXO, M1-EXO, and antagomir-155-5p transfected M1-EXO. White arrowheads: activated microglia. Scale bar, 20 μm. (G) The percentages of activated PMGs per field with multipolar and ameboid shapes were quantified (n = 6, **P<0.01). (F) Percentage of CD11b^+^ CD86^+^ PMGs among the four groups. (I) Statistical analysis of the percentage of CD11b- and CD86-positive cells of PMGs (n = 6, ***P<0.001). (J) Representative flat mount images stained with Iba1 (red) in different treatment groups. Scale bar, 50 μm. (K) Quantification of the cell number per field in different treatment groups (n ≥ 6, ***P<0.001, **P<0.01). PMGs: primary microglia; EXO: exosomes.

Emerging evidence has shown that exosomes are enriched with miRNAs and deliver miRNAs further to regulate the function of recipient cells 46,47. To elucidate the specific mechanism of microglial cell activation by M1-EXO, we performed miRNA sequencing on exosomes derived from BV2 cells under resting and M1-activated conditions. As shown in the volcano plot (Figure 7A), by setting a cutoff of FDR<0.05 and |log2FC|>1, we found 103 differentially expressed miRNAs in M1-EXO compared with M0-EXO, including 16 upregulated and 87 downregulated miRNAs. The intra- and intergroup differences in the identified miRNAs were further visualized using a heatmap (Figure 7B).

Moreover, by using qRT‒PCR, we found that only the level of miR-155-5p in both M1 BV2 cells and M1-EXO was significantly higher than the respective controls (cells: miR-155-5p, 25.067±8.429-fold, **P<0.01; exosomes: miR-155-5p, 64.187±7.018-fold, ***P<0.001) (Figure 7C). However, the changes in miR-146b-5p, miR-181d-3p, and miR-5121 were not consistent between the cells and exosomes (cells: miR-146b-5p, 0.627±0.100-fold, *P*>0.05; miR-181d-3p, 8.403±0.653-fold, ***P<0.001; miR-5121, 2.586±0.0.332-fold, **P<0.01; exosomes: miR-146b-5p, 0.260±0.046-fold, ***P<0.001; miR-181d-3p, 1.883±0.794-fold, *P*>0.05; miR-5121, 1.274±0.253-fold, *P*>0.05). Thus, miR-155-5p might play a key role in exosomes and be further tested in our present study.

To further clarify the exact role of miR-155-5p in exosomes derived from BV2 cells, the expression patterns of miR-155-5p in exosomes were modulated, and the roles of modified exosomes on PMGs were observed. Firstly, we overexpressed miR-155-5p by agomir transfection in the M0-EXO, which was originally low in miR-155-5p expression (ago-miR-155-5p group). Secondly, we suppressed miR-155-5p by antagomir transfection in the M1-EXO, which originally highly expressed miR-155-5p (antago-miR-155-5p group). Then, we identified the M1 activation profile of the PMGs treated with the modified EXO respectively. Three M1 phenotype markers (iNOS, Il1β, CD86) were utilized to identify the M1 activation profile. Our data showed that miR-155-5p overexpression in M0-EXO significantly caused dramatic morphological changes (agomiR-NC: 7.2±3.9%; agomiR-155-5p: 29.1±7.8%, ***P<0.001) (Figure 7F-G) and facilitated M1 polarization of resting PMGs (iNOS: agomiR-NC: 0.328±0.059; agomiR-155-5p: 0.793±0.164; **P<0.01; Il1β: agomiR-NC: 0.683±0.069; agomiR-155-5p: 0.944±0.102, *P<0.05; CD11b^+^ CD86^+^: agomiR-NC: 12.3±1.9%; agomiR-155-5p: 30.7±4.6%, ***P<0.001) (Figure 7D-E, Figure 7H-I, Figure S3A). Notably, when miR-155-5p was downregulated by antagomiR-155-5p in M1-EXO, the ability of M1-EXO to promote M1 polarization was significantly suppressed, which was reflected by decreased morphological abnormalities (antagomiR-NC: 57.6±10.9%; antagomiR-155-5p: 29.8±10.3%, **P<0.01) (Figure 7F-G) and activated markers (iNOS: antagomiR-NC: 0.883±0.021; antagomiR-155-5p: 0.700±0.087; *P<0.05; Il1β: antagomiR-NC: 0.847±0.047; antagomiR-155-5p: 0.373±0.145, **P<0.01; CD11b^+^ CD86^+^: antagomiR-NC: 78.9±6.7%; antagomiR-155-5p: 48.4±7.0%, ***P<0.001) (Figure 7D-E, Figure S3B).

Next, to confirm miR-155-5p bioactivity *in vivo*, exosomes transfected with agomiR or antagomiR were injected into the vitreous chamber. The results of retinal flat mounts showed that exogenous miR-155-5p supplementation facilitated M0-EXO to induce PMG activation, and the downregulation of miR-155-5p from M1-EXO alleviated microglial activation (agomiR-NC: 18.14±3.934; agomiR-155-5p: 27.00±5.715, **P<0.01; antagomiR-NC: 65.57±11.86; antagomiR-155-5p: 33.29±6.945, ***P<0.001) (Figure 7J-K). Therefore, exosomal miR-155-5p could regulate the polarization states of microglia, and it might be a crucial molecule for exosome-mediated microglial activation.

### Exosomal miR-155-5p is involved in microglial activation by regulating the Socs1/NFκB pathway

**Figure 8:**
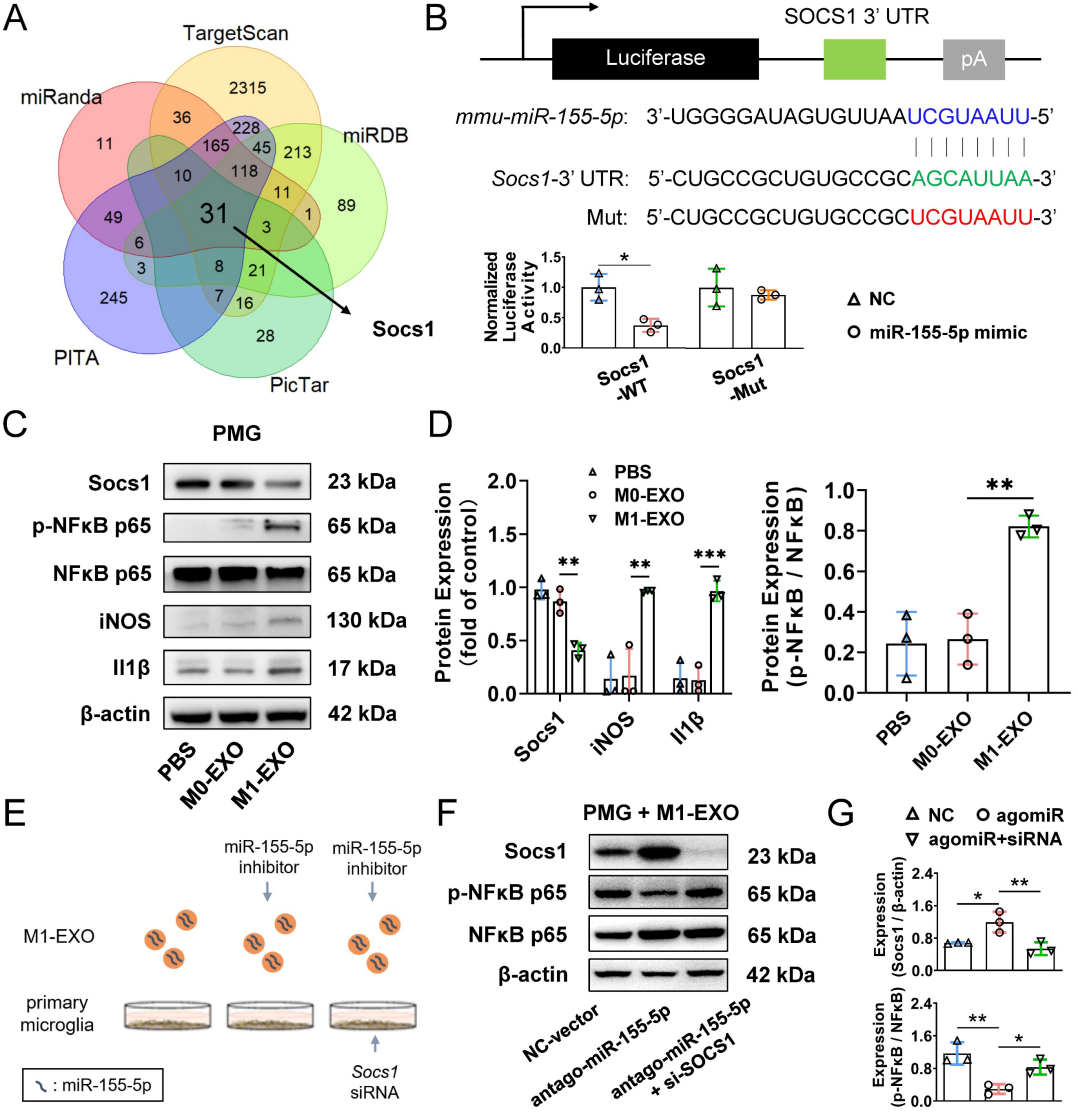
** Exosomes miR-155-5p promotes microglia activation by regulating the NFκB pathway via targeting Socs1.** (A) The Venn diagram shows the overlap of the data from five miRNA databases. (B) Dual-luciferase reporter assays were conducted to confirm a direct interaction between miR-155-5p and Socs1. Upper panel: sequence alignment of miR-155-5p and its putative-binding sites in Socs1 mRNA 3'-UTR. The mutant type was also constructed. Lower panel: luciferase activity was presented as relative luciferase activity normalized to activity of their respective negative control (n = 3, *P<0.05). (C) Western blot analysis indicates that M1-EXO treatment downregulated Socs1 expression, and subsequently upregulated phosphorylated NFκB and M1-activated marker genes expression in the PMGs. (D) Relative quantification of protein expression in PMGs (n = 3, ***P<0.001, **P<0.01, *P<0.05). (E) Schematic diagram of loss-of-functions. PMGs treated with M1-EXO, antagomir-155-5p transfected M1-EXO, or transfected M1-EXO combined with Socs1 knockdown for 36h. (F) Western blot results show the changing protein level of Socs1 and phosphorylated NFκB after antagomir-155-5p transfected M1-EXO treatment alone or in combination with Socs1 knockdown. (G) Relative quantification of protein expression in PMGs (n = 3, ***P<0.001, **P<0.01). PMGs: primary microglia; EXO: exosomes; WT: wild type; Mut: mutant.

To explore the mechanism underlying the activation and inflammatory switch of resting microglia by miR-155-5p, we predicted the potential target genes of miR-155-5p using bioinformatics analysis. Five databases (miRanda, miRDB, PITA, TargetScan, and PicTar) were utilized to obtain 32 intersected mRNAs (Figure 8A). The overlapping candidate mRNAs were further subjected to functional enrichment analysis to identify inflammation-related pathways. Based on a series of bioinformatics analyses, Socs1 was supposed to be the critical target of miR-155-5p because it mediates the signaling of immunity and inflammation (Table S2). Hence, to confirm the results from bioinformatics analysis, a dual-luciferase reporter plasmid system containing the 3'-UTR of Socs1 mRNA was constructed. Luciferase reporter plasmids and miR-155-5p mimics or scramble mimics were cotransfected into PMGs. Analysis of luciferase activities showed that miR-155-5p interacted with Socs1 3'-UTR directly and decreased the expression of Socs1 (NC+WT: 1.000±0.219; mimic+WT: 0.372±0.107, *P<0.05) (Figure 8B); thus, the regulation of Socs1 might be the key for deciphering the mechanism between exosomal miR-155-5p and retinal inflammation.

Previous studies have shown that Socs1 limits NFκB signaling and nuclear NFκB activity by interacting with the NFκB subunit p65, and the activation of the NFκB family of transcription factors is a crucial step in regulating microglial activation and proinflammatory molecules expression 48,49. Therefore, Socs1 might be involved in M1-EXO-mediated cell activation by regulating NFκB, which was confirmed by Western blot assay. As shown in Figure 8C-D, M1-EXO significantly inhibited the expression of Socs1, followed by the upregulation of phosphorylated NFκB and two proinflammatory markers (iNOS, Il1β) (Socs1: PBS: 0.979±0.093, M0-EXO: 0.868±0.113, M1-EXO: 0.410±0.069, *F_2,6_*=31.44, ***P<0.001; iNOS: PBS: 0.138±0.199, M0-EXO: 0.170±0.251, M1-EXO: 0.963±0.011, *F_2,6_*=19.10, **P<0.01; Il1β: PBS: 0.146±0.151, M0-EXO: 0.127±0.126, M1-EXO: 0.966±0.097, *F_2,6_*=43.04, ***P<0.001; NFκB/p-NFκB: PBS: 0.243±0.157, M0-EXO: 0.266±0.126, M1-EXO: 0.822±0.053, *F_2,6_*=22.29, **P<0.01).

To further demonstrate that the proinflammatory role of M1-EXO was mediated by Socs1 and the downstream transcription factor NFκB, loss-of-function experiments were conducted (Figure 8E). As presented in Figure 8F, M1-EXO transfected with antagomiR-155-5p significantly promoted Socs1 expression and downregulated the expression of phosphorylated NFκB in PMGs. Knocking down Socs1 via siRNA transfection into PMGs also significantly alleviated the inhibitory effects of antagomiR-155-5p compared with empty vector transfection. (Socs1: NC: 0.679±0.015, antagomiR: 1.192±0.257, antagomiR+siRNA: 0.537±0.157, F2,6=11.76, **P<0.01; NFκB/p-NFκB: NC: 1.164±0.273, antagomiR: 0.293±0.119, antagomiR+siRNA: 0.829±0.182, *F_2,6_*=14.26, **P<0.01) (Figure 8F-G). Therefore, the knockdown of Socs1 could mimic the similar proinflammatory effects of exosomal miR-155-5p. These results suggested that Socs1, the target of miR-155-5p, plays a crucial role in microglial activation and retinal inflammation.

### Transcription factor Irf1 promotes cellular and exosomal miR-155-5p in microglia

**Figure 9:**
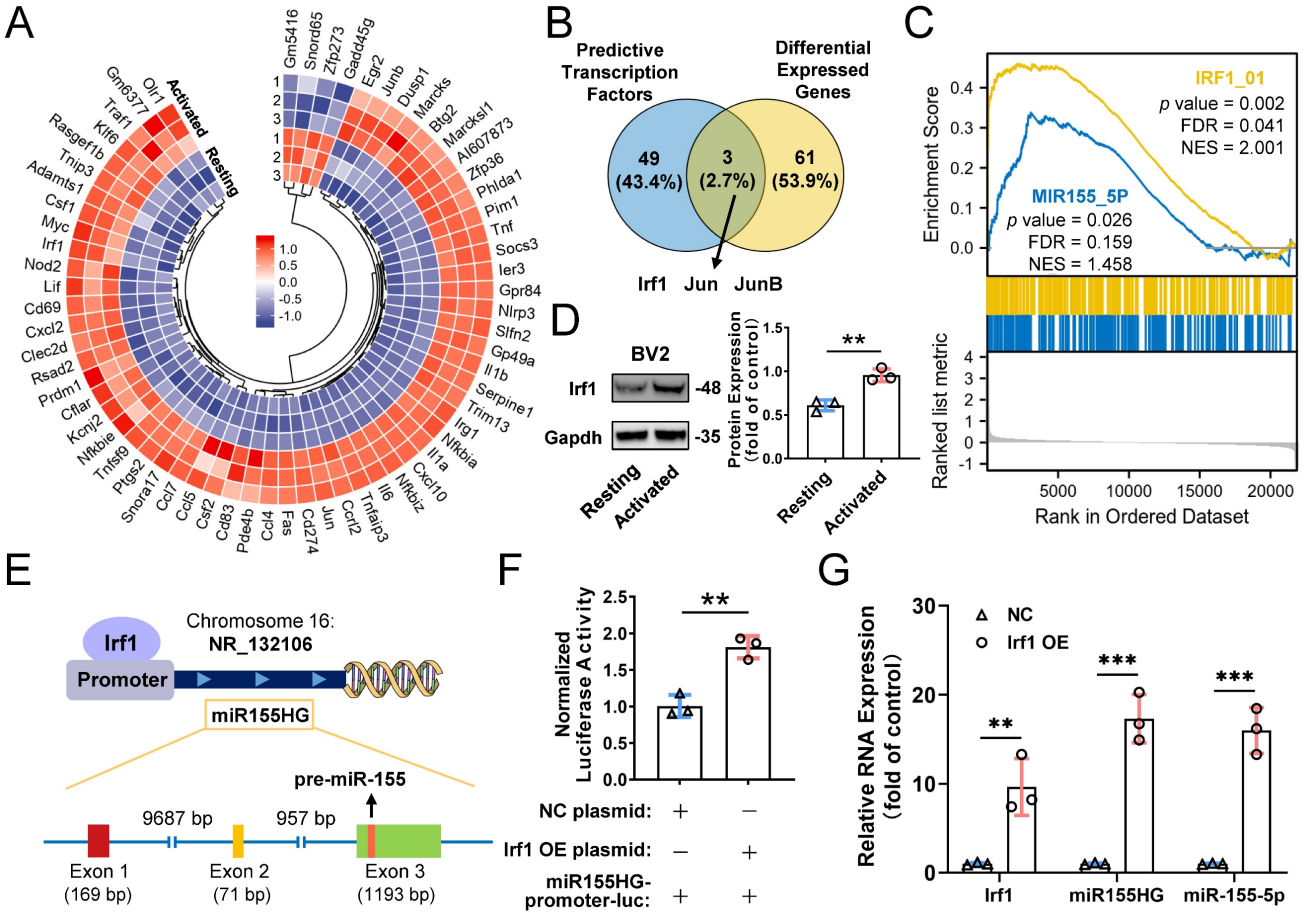
**Transcription factors Irf1 promotes the expression of miR-155-5p in M1-activated microglia.** (A) Heatmap of differential mRNAs between resting and M1 activated BV2 cells based on mRNA sequencing. (B) Overlapping results among the differential mRNA from mRNA sequencing and potential direct transcription factors of miR-155-5p predicted by JASPAR database. (C) Gene set enrichment analysis of Irf1 and miR-155-5p related signaling between resting and activated microglia. (D) Representative gel images and the densitometry quantification of Irf1 (n = 3, **P<0.01). (E) The diagram shows the binding sites of Irf1 in the specific sequence of the host gene of miR-155-5p (miR155HG). (F) Relative luciferase activity of miR155HG (including miR-155-5p promoter) in BV2 cells after the co-transfection of Irf1 overexpression or negative control plasmids and miR-155-5p-promoter luciferase plasmids (n = 3, **P<0.01). (G) The RNA levels of Irf1, miR155HG, and miR-155-5p in Irf1 OE and negative control groups were determined by qRT-PCR. NC: negative control; OE: over-expression; qRT-PCR: quantitative real-time reverse transcription-polymerase chain reaction.

Based on our results mentioned above, we confirmed that the cellular miR-155-5p level was increased in M1-activated microglia and thereby caused an increase in miRNA levels in exosomes, which ultimately propagated activated signals and inflammatory responses among microglia. However, how exactly miR-155-5p is increased in activated microglia is still elusive. Mounting evidence indicates that transcription factors (TFs) can directly or indirectly trigger the expression of miRNAs 50,51. To ascertain the potential TFs responsible for the upregulation of miR-155-5p, mRNA sequencing between resting BV2 cells and activated BV2 cells was performed to screen differentially expressed genes **(Figure 9A)**. Moreover, intersection analysis of the differentially expressed genes and potential transcription factors predicted by the JASPAR database showed that three candidates might be responsible for the expression of miR-155-5p** (Figure 9B)**. After GSEA of these candidates, we found that transcription factor Irf1-related pathways were greatly enriched in activated microglia compared to resting cells **(Figure 9C)**. Moreover, the protein expression of Irf1 was significantly increased in activated BV2 microglia compared to resting BV2 microglia (Resting: 0.610±0.062; Activated: 0.955±0.074, **P<0.01)** (Figure 9D)**.

It is already known that miR-155-5p is processed from the miR155 host gene (miR155HG) 52. To confirm that the transcription factor Irf1 binds to the promoters of miR155HG and subsequently promotes the expression of miR-155-5p** (Figure 9E)**, a miR155HG promoter-luciferase reporter plasmid system was constructed, and it was cotransfected with Irf1 overexpression plasmids into BV2 cells. Luciferase activities were detected after transfection. **Figure 9F** shows that Irf1 could activate the promoters of miR155HG (NC: 1.007±0.151; Irf1 OE: 1.812±0.153, **P<0.01). Moreover, overexpression of Irf1 significantly promoted the expression of both miR155HG and miR-155-5p in BV2 microglia (miR155HG: 17.315±2.728-fold, ***P<0.001; miR-155-5p: 15.983±2.588-fold, ***P<0.001)** (Figure 9G)**. All these results demonstrated that upregulated Irf1 directly binds the specific DNA sequences of miR155HG and promotes the expression of miR-155-5p in activated microglia.

## Discussion

Neovascular retinal diseases have a tremendous effect on the quality of life 53. Therefore, it is essential to understand their pathophysiology and discover therapeutic strategies to interfere with their progression. In the present study, our *in vitro* results demonstrated that M1 microglia activate resting microglia and promote the proliferation and tube formation of endothelial cells through the M1 microglial exosome-mediated signaling pathway. Moreover, this evidence was confirmed *in vivo*. Exosomes derived from M1 microglia enhanced inflammation and promoted the activation of resting microglia and angiogenesis through intravitreal injection. Furthermore, our study elucidated the molecular mechanism of M1 microglial exosome-mediated angiogenesis, in which M1 microglial exosomes induce M0 microglial activation via exosomal miR-155-5p. Exosomal miR-155-5p is involved in microglial activation and propagates an inflammatory cascade by regulating the Socs1/NFκB pathway, and the transcription factor Irf1 promotes cellular and exosomal miR-155-5p in microglia. Thus, so far as we are aware, this is the first study that revealed a novel mechanism of M1 microglial exosome-mediated activation and migration of resting microglia, the inflammatory cascade, and angiogenesis in the retina.

The strength of this study was based on a publicly available single-cell RNA sequencing dataset (accession no. GSE152928) and bioinformatics approaches, which could save much experimental consumption (time and money). Traditional methods require years of work in the lab. According to bioinformatics analysis of the active state of M1 microglia** (Figure 1C)** and receptor‒ligand pairs among various cells **(Figure 1D-E)**, we deduced that intercellular communication from M1 microglia toward M0 microglia was increased. Moreover, according to exosomes expressing markers of Cd63 and Sdcbp, M1 microglia-derived exosomes probably mediated the above process **(Figure 1F-G)**. Therefore, these analysis results provide evidence to support our hypothesis that M1 microglia might activate M0 microglia through the exosome-mediated signaling pathway.

Moreover, our results, both* in vitro* and *in vivo*, strongly support the results of bioinformatics approaches. First, M1 microglial exosomes promote primary resting microglial activation *in vitro*. The relative inflammatory factors were significantly increased in activated primary resting microglia **(Figure 3)**. Accordingly, M1 microglial exosomes delivered by intravitreal injection were absorbed by retinal microglia *in vivo*, and the number of activated M1 microglia was significantly increased in the retina compared to that of M0 microglial exosomes** (Figure 5)**. The factors involved in angiogenesis, Sdf1 and Cxcr4, were strongly expressed, and proliferation of endothelial cells was observed in the retina after treatment with M1 microglial exosomes** (Figure 6A-B)**. Minocycline, an inhibitor of microglial activation, significantly alleviated the increase in endothelial cells induced by M1 microglial exosomes **(Figure 6C-E)** 44,45,54. Thus, we provide evidence, for the first time, that M1 microglial exosomes eventually cause abnormal endothelial cell proliferation in the retina, a fundamental early step in the angiogenic response 31, and this process is based on retinal inflammation, which plays a critical role in most neovascular ocular diseases 12.

More importantly, our discovery could account for the perplexity of the activation and accumulation of microglia in the damaged lesion in previous studies. Previous studies have shown that resting microglia strongly inhibit endothelial cell proliferation and even alleviate retinal neovascularization at early stages 31,55. Resting microglia in the retina could exert an active anti-angiogenic effect, allowing the retina to maintain homeostasis without suffering from neovascularization. However, how is this homeostasis disrupted in the development of neovascular retinal diseases? With an example of the laser-induced CNV model, our data suggest that laser damage induced microglia activation, and activated microglia in the site of injury might produce exosomes. Subsequently, nearby resting microglia absorbed these exosomes, being activated and migrating to the lesions in the laser-induced CNV model, and ultimately exerted proangiogenic rather than antiangiogenic effects. Our findings could have important implications for targeting activated microglia-derived exosomes to hinder the progression of neovascular retinal diseases.

Furthermore, we reveal the molecular mechanisms of M1 microglial exosome-mediated proangiogenesis. The concentration of miR-155-5p was significantly higher in M1-activated cells and exosomes than in the respective controls **(Figure 7C)**. miR-155-5p has been recognized as a proinflammatory factor, and it has been implicated in the M1 polarization of macrophages in various inflammatory diseases 56,57. A previous study demonstrated that dysregulated miR-155 increases the inflammatory load and microglial activation under retinal ischemic conditions, similar to our study. However, how miR-155-5p is transmitted between cells and affects target cells during retinal inflammation remains unclear 58. To our knowledge, this study offers the first demonstration that exosomes function as a bridge between M1 microglia and the spread of retinal inflammation by transferring miR-155-5p, further devastating the retina's vascular homeostasis.

In addition, based on a series of bioinformatics and experimental methods, we revealed the upstream-downstream molecular mechanisms of miR-155-5p. First, Socs1 was identified as the downstream target of exosomal miR-155-5p, which is supported by previous findings 59. Several studies have indicated Socs1 as an inflammatory suppressor gene in ocular disorders, including diabetic retinopathy and uveitis 60,61. Moreover, Socs1 negatively regulates macrophage/microglia activation 62. However, many issues remain to be resolved in the complex environment of the retina, especially the exact mechanisms governing microglial Socs1 expression during retinal inflammation. The current study represents a continuation of previous studies and focuses on identifying a vital mediator of these phenotypes - microglial exosomes. We confirmed that the miR-155-5p/Socs1/NFκB axis mediated by M1 exosomes plays an essential role in the spread of retinal inflammation.

Currently, how the expression of miRNAs is regulated under specific contexts is another crucial subject in miRNA studies 63. In addition to the downstream target gene of miR-155-5p, we further revealed that the transcription factor Irf1 was an upstream regulator of miR-155-5p. Irf1 drives the expression of miR-155-5p in microglia by directly binding to the promoter of miR155HG, the host gene of miR-155-5p. Previous studies also supported this finding. There have been some reports on Irf1 and macrophage/microglia polarization. On the one hand, Carey *et al.* demonstrated that Irf1 and NFκB synergistically enhance proinflammatory molecules expression, which ultimately polarizes macrophages to the M1 phenotype 64. On the other hand, it has been claimed that Irf1 could act as a blocker of the Il4 promoter, thereby inhibiting macrophage polarization to the M2 phenotype 65. However, there was a report slightly different from our study. Sahmatova* et al.* described the interconnectedness of Irf1 and miR-155. They suggested that Irf1 was only a downstream target gene of miR-155 and was negatively correlated with miRNA expression, whereas our data indicated that Irf1 was located upstream of miR-155-5p and its precursor in microglia 66. This divergence may be attributed to different tissues and disease states. Thus, our study reveals the mechanisms from transcription to delivery, in which Irf1/miR-155-5p/Socs1 is involved in M1 microglia-derived exosomes promoting the activation of resting microglia and amplifying proangiogenic effects in the retina.

Of course, there is a limitation in the present study that the complete process of pathological neovascularization was not observed in the *in vivo* experiments, and only abnormal endothelial cell proliferation was found. This might be attributed to the resistance of healthy mice to pathological substances, and increasing the concentration of proinflammatory exosomes might achieve more desirable angiogenic results. However, the pathological process developed slowly in this model, which is consistent with the actual process in patients. Thus, this model is a promising pathological animal model. More investigation is required in our next study.

**Figure 10:**
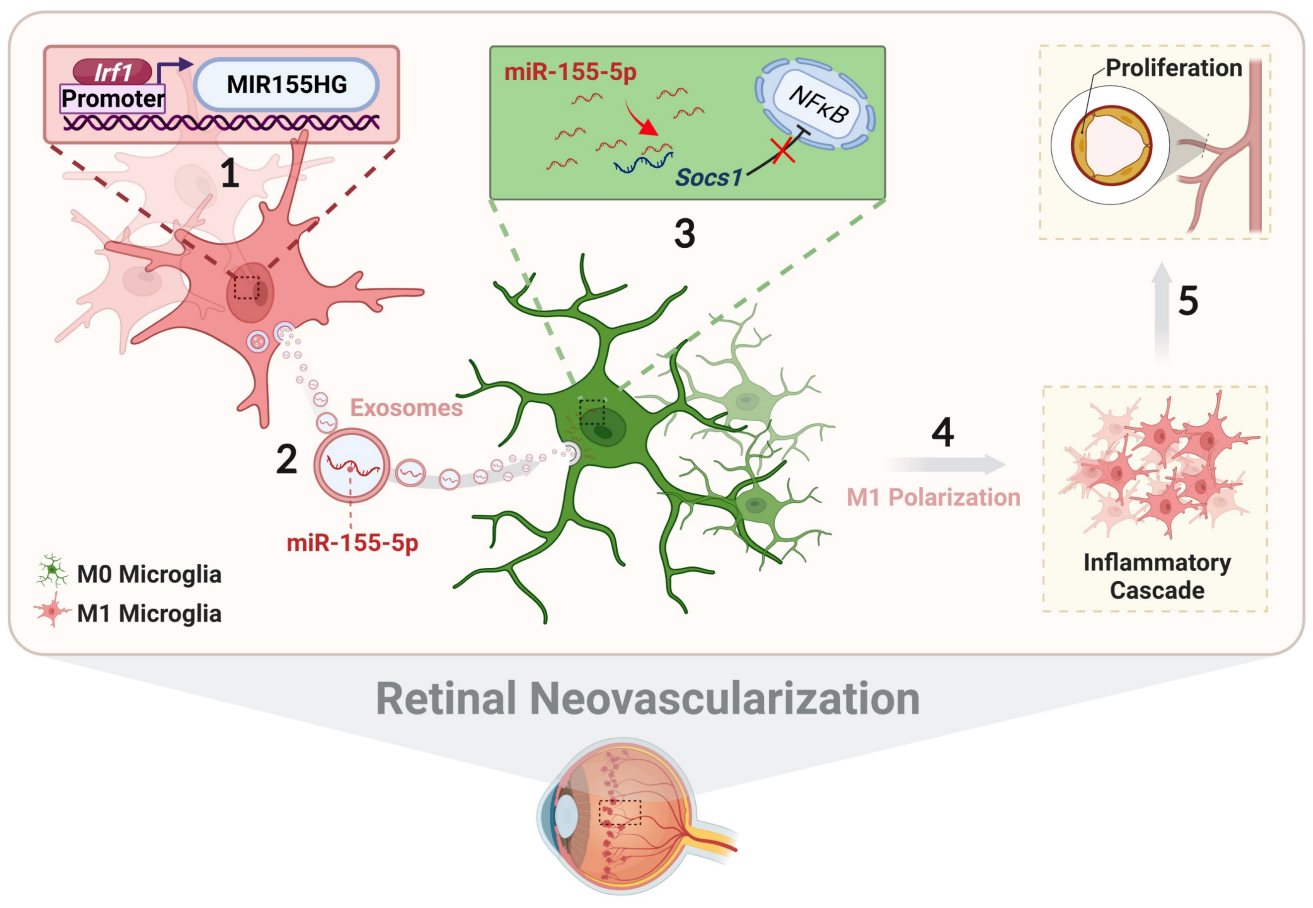
** The schematic diagram illustrates how Irf1 mediated exosomal miR-155-5p in M1 microglia regulates Socs1 expression to promote the activation of resting microglia and amplify pro-angiogenic effects.** (1) Upregulated Irf1 directly binds the specific DNA sequences of miR155HG and promotes the expression of miR-155-5p in activated microglia. (2) Exosomal miR-155-5p from M1-activated microglia is endocytosed by resting microglia. (3) Exosomal miR-155-5p activates the NFκB pathway and microglia activation state via selectively suppressing Socs1. (4) Increased and aggregated M1 microglia cause inflammatory cascade and (5) amplify pro-angiogenic effects, ultimately inducing retinal abnormal endothelial cell proliferation.

In conclusion, we systematically illustrate the relationship among exosomal miR-155-5p, microglial polarization, and neovascularization, including intercellular communication between various types of microglia in the complex retinal microenvironment during angiogenesis. As presented in **Figure 10**, activated microglia-derived exosomes transmitted polarization ability to resting microglia by transferring miR-155-5p. Increasing miR-155-5p could suppress Socs1 to activate the NFκB pathway, ultimately promoting microglial activation and the abnormal proliferation of vascular endothelial cells. In addition, Irf1 was upregulated, efficiently driving the expression of miR-155-5p in activated microglia, thus leading to an increase in the tendency for miR-155-5p to be encapsulated by exosomes. Therefore, exosomal miR-155-5p derived from M1 microglia could cause cascading and amplification effects on retinal inflammation and serve as the indirect driving factor of retinal neovascularization. This study not only elucidates the mechanism and contributes to novel, targeted, and potential therapeutic strategies for clinical retinal neovascularization but also develops a new pathological animal model.

## Supplementary Material

Supplementary figures and tables.

## Abbreviations

EXO: exosomes
miRNA: microRNAs
CNV: choroidal neovascularization
TFs: transcription factors
CON: control
OIR: oxygen-induced retinopathy
ECs: endothelial cells
TEM: transmission electron microscopy
NTA: transmission electron microscopy
PMGs: primary microglia
HUVECs: human umbilical vein endothelial cells
CM: conditioned medium
GCL: ganglion cell layer
INL: inner nuclear layer
ONL: outer nuclear layer
Mino: minocycline
qRT-PCR: quantitative real-time reverse transcription-polymerase chain reaction
NC: negative control
OE: over-expression
WT: wild type
Mut: mutant
t-SNE: t-distributed scholastic neighbor embedding
