# Supplementary material for: M1 Microglia-derived Exosomes Promote Activation of Resting Microglia and Amplifies Proangiogenic Effects through Irf1/miR-155-5p/Socs1 Axis in the Retina
Source: Int J Biol Sci. 2023 Mar 21;19(6):1791–812. doi: 10.7150/ijbs.79784 (PMC10092772; doi:10.7150/ijbs.79784)
Supplement: Supplementary file 1 — Supplementary figures and tables. [file ijbsv19p1791s1.pdf]

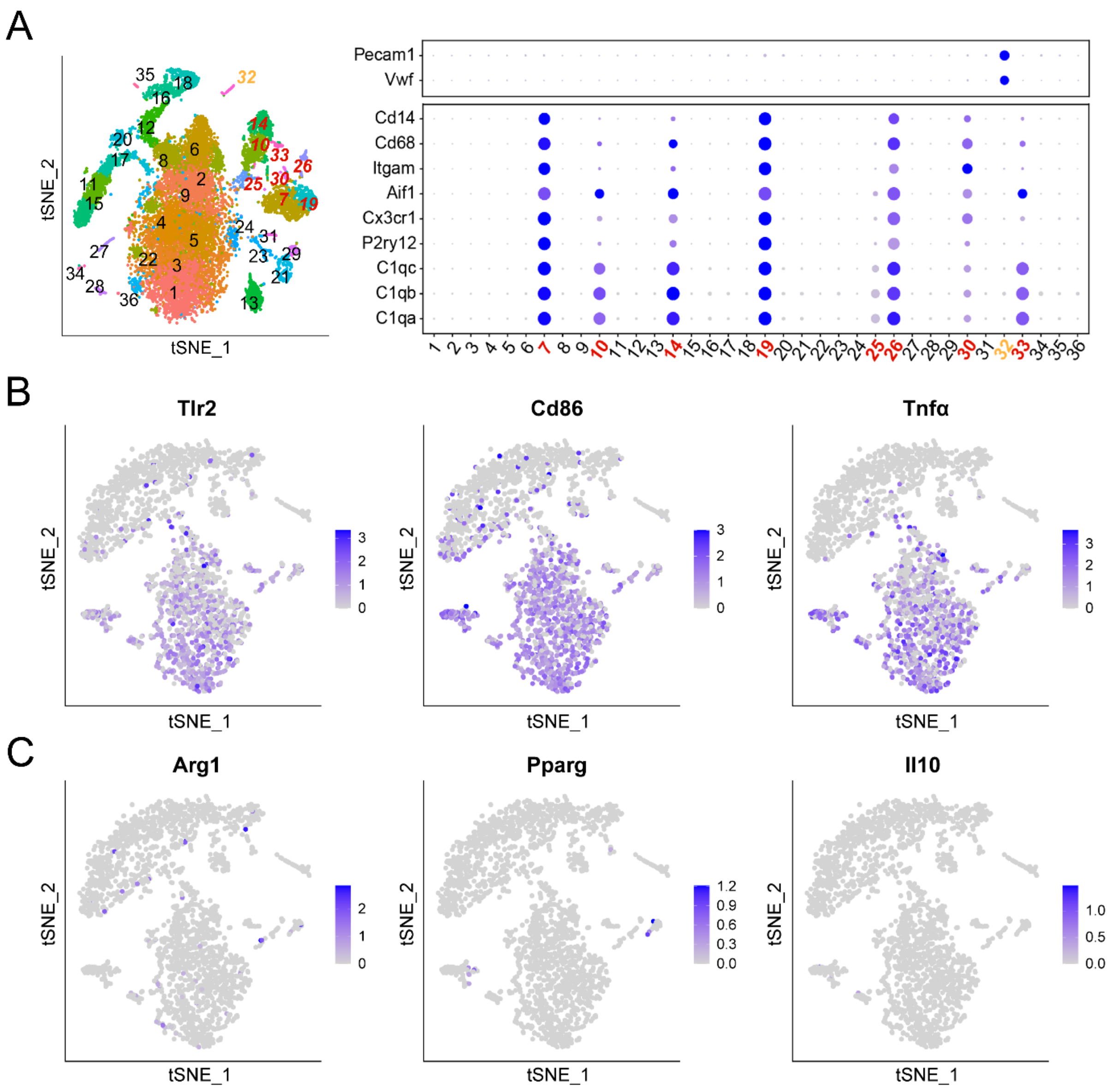

Figure S1. Classification of retinal cell types from mouse OIR models. (A) Left panel: unsupervised tSNE clustering of the 15185 cells yielded 36 clusters. Red represents microglia/macroglia, and yellow represents endothelial cells. Right panel: feature expression heatmap showing expression patterns of the markers of microglia/macrophage and endothelial cells across 36 retinal cell clusters. The size of each circle depicts the percentage of cells expressing the marker within the cluster. (B) tSNE clustering overlaid with heatmaps of genes previously reported to be enriched in M1 type microglia (Tlr2, CD86, Tnfα) and (C) Arg1, Pparg, Il10 for M2 type microglia. Cells that highly express these genes are colored in purple, while cells with low expression are colored in grey.

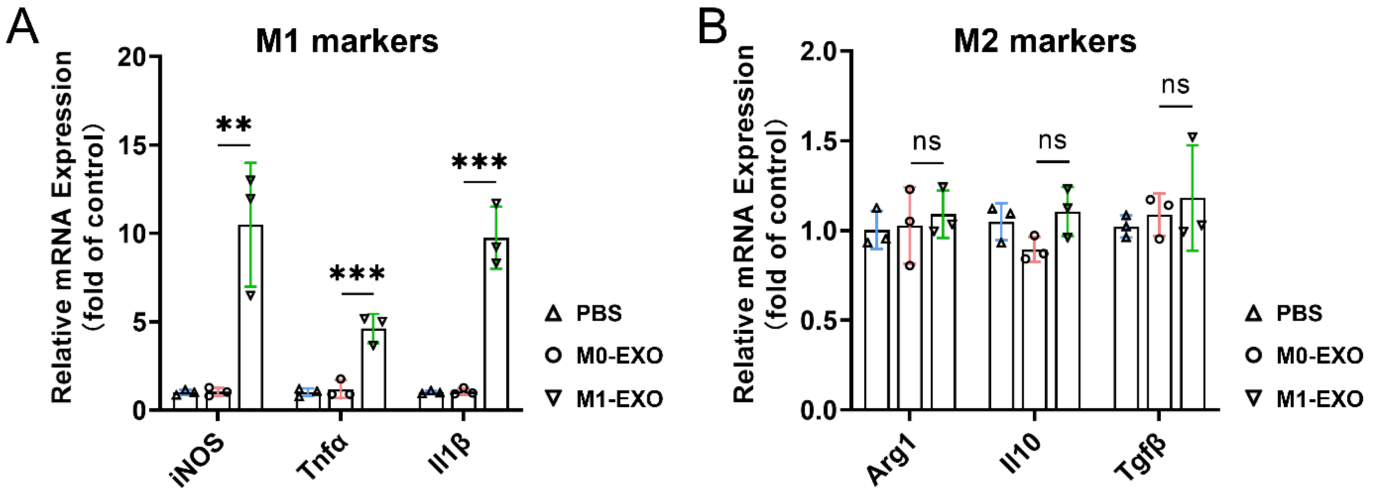

Figure S2. M1 microglial exosomes modulate resting microglia to M1 phenotype rather than M2 phenotype. (A) The mRNA levels of M1 phenotype markers (iNOS, Tnfα, Il1β) after M1-EXO treatment were determined by qRT-PCR (n = 3, ***P*<0.01, ****P*<0.001). (B) The mRNA levels of M2 phenotype markers (Arg1, Il10, Tgfβ) after M1-EXO treatment were determined by qRT-PCR. qRT-PCR: quantitative real-time reverse transcription-polymerase chain reaction.

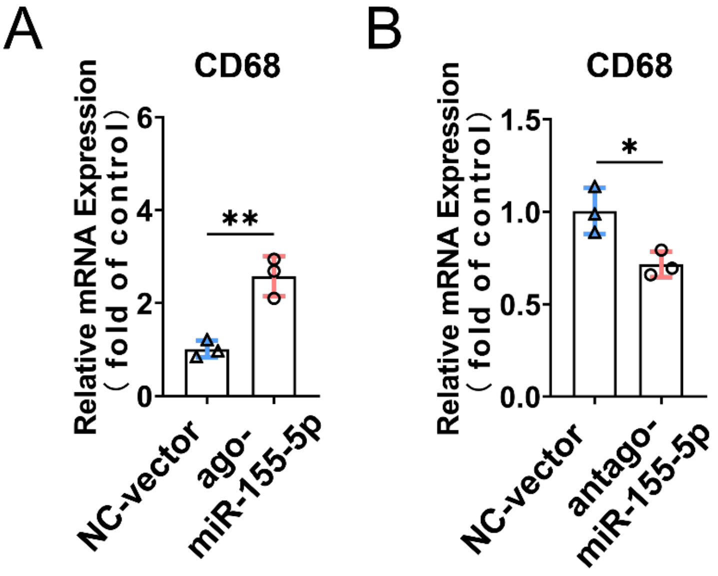

Figure S3. Exosomal miR-155-5p regulate the M1 polarization states of microglia. (A) The mRNA levels of CD68 after treatment with M0-EXO and agomir-155-5p transfected M0-EXO. (n = 3, ***P*<0.01). (B) The mRNA levels of CD68 after treatment with M1-EXO and antagomir-155-5p transfected M1-EXO. (n = 3, **P*<0.05).

Table S2. Socs1- related gene ontology annotation.

ONTO-LOGY	ID	Description	BgRatio	pvalue	qvalue	gene
BP	GO:1903708	positive regulation of hemopoiesis	208/23210	1.93E-07	5.44E-05	Inpp5d / Myb / Socs1 / Hif1a / Shh / Gnas
BP	GO:0046632	alpha-beta T cell differentiation	112/23210	2.59E-07	5.48E-05	Myb / Socs1 / Satb1 / Itk / Shh
BP	GO:0046631	alpha-beta T cell activation	157/23210	1.38E-06	0.0002	Myb / Socs1 / Satb1 / Itk / Shh
BP	GO:1902107	positive regulation of leukocyte differentiation	168/23210	1.93E-06	0.00023	Inpp5d / Myb / Socs1 / Shh / Gnas
BP	GO:0030098	lymphocyte differentiation	395/23210	7.98E-06	0.00063	Inpp5d / Myb / Socs1 / Satb1 / Itk / Shh
BP	GO:1903706	regulation of hemopoiesis	407/23210	9.46E-06	0.00063	Inpp5d / Myb / Socs1 / Hif1a / Shh / Gnas
BP	GO:0045621	positive regulation of lymphocyte differentiation	108/23210	9.63E-06	0.00063	Inpp5d / Myb / Socs1 / Shh
BP	GO:0030217	T cell differentiation	269/23210	1.91E-05	0.00083	Myb / Socs1 / Satb1 / Itk / Shh
BP	GO:1903037	regulation of leukocyte cell-cell adhesion	287/23210	2.60E-05	0.00091	Myb / Socs1 / Socs6 / Rela / Shh
BP	GO:0042110	T cell activation	491/23210	2.73E-05	0.00091	Myb / Socs1 / Satb1 / Socs6 / Itk / Shh
BP	GO:1902105	regulation of leukocyte differentiation	294/23210	2.92E-05	0.00091	Inpp5d / Myb / Socs1 / Shh / Gnas
BP	GO:0046638	positive regulation of alpha-beta T cell differentiation	49/23210	3.11E-05	0.00094	Myb / Socs1 / Shh
BP	GO:0051250	negative regulation of lymphocyte activation	152/23210	3.70E-05	0.00102	Inpp5d / Socs1 / Socs6 / Shh
BP	GO:0007159	leukocyte cell-cell adhesion	319/23210	4.30E-05	0.0011	Myb / Socs1 / Socs6 / Rela / Shh

BP	GO:0019221	cytokine-mediated signaling pathway	341/23210	5.91E-05	0.00139	Socs1 / Rps6ka5 / Trim44 / Ikbke / Rela
BP	GO:0002695	negative regulation of leukocyte activation	182/23210	7.45E-05	0.00166	Inpp5d / Socs1 / Socs6 / Shh
BP	GO:0045619	regulation of lymphocyte differentiation	182/23210	7.45E-05	0.00166	Inpp5d / Myb / Socs1 / Shh
BP	GO:0046637	regulation of alpha-beta T cell differentiation	68/23210	8.32E-05	0.00172	Myb / Socs1 / Shh
BP	GO:0046635	positive regulation of alpha-beta T cell activation	69/23210	8.69E-05	0.00175	Myb / Socs1 / Shh
BP	GO:0050866	negative regulation of cell activation	203/23210	0.000114	0.00213	Inpp5d / Socs1 / Socs6 / Shh
BP	GO:1903039	positive regulation of leukocyte cell-cell adhesion	203/23210	0.000114	0.00213	Myb / Socs1 / Rela / Shh
BP	GO:0022407	regulation of cell-cell adhesion	395/23210	0.000118	0.00213	Myb / Socs1 / Socs6 / Rela / Shh
BP	GO:0043367	CD4-positive, alpha-beta T cell differentiation	81/23210	0.00014	0.00242	Myb / Socs1 / Satb1
BP	GO:0006650	glycerophospholipid metabolic process	225/23210	0.000169	0.0028	Fgf7 / Inpp5d / Socs1 / Socs6
BP	GO:0043374	CD8-positive, alpha-beta T cell differentiation	16/23210	0.000179	0.00286	Socs1 / Satb1
MF	GO:0005159	insulin-like growth factor receptor binding	14/22710	0.000152	0.00293	Socs1 / Gnas
MF	GO:0046935	1-phosphatidylinositol-3-kinase regulator activity	14/22710	0.000152	0.00293	Socs1 / Socs6
BP	GO:0022409	positive regulation of cell-cell adhesion	242/23210	0.000223	0.00325	Myb / Socs1 / Rela / Shh
BP	GO:0045582	positive regulation of T cell differentiation	95/23210	0.000225	0.00325	Myb / Socs1 / Shh

BP	GO:0035710	CD4-positive, alpha-beta T cell activation	100/23210	0.000261	0.00357	Myb / Socs1 / Satb1
BP	GO:1902106	negative regulation of leukocyte differentiation	102/23210	0.000277	0.00372	Inpp5d / Socs1 / Shh
BP	GO:0046634	regulation of alpha-beta T cell activation	104/23210	0.000293	0.00382	Myb / Socs1 / Shh
MF	GO:0035014	phosphatidylinositol 3-kinase regulator activity	19/22710	0.000284	0.00412	Socs1 / Socs6
BP	GO:0001959	regulation of cytokine-mediated signaling pathway	108/23210	0.000328	0.00414	Socs1 / Trim44 / Ikbke
BP	GO:0060759	regulation of response to cytokine stimulus	118/23210	0.000425	0.00514	Socs1 / Trim44 / Ikbke
BP	GO:0050868	negative regulation of T cell activation	119/23210	0.000436	0.00519	Socs1 / Socs6 / Shh
BP	GO:0036037	CD8-positive, alpha-beta T cell activation	27/23210	0.000519	0.00563	Socs1 / Satb1
BP	GO:0046639	negative regulation of alpha-beta T cell differentiation	27/23210	0.000519	0.00563	Socs1 / Shh
BP	GO:0050863	regulation of T cell activation	304/23210	0.000529	0.00567	Myb / Socs1 / Socs6 / Shh
BP	GO:0046488	phosphatidylinositol metabolic process	129/23210	0.000551	0.00569	Inpp5d / Socs1 / Socs6
BP	GO:1903038	negative regulation of leukocyte cell-cell adhesion	131/23210	0.000577	0.00569	Socs1 / Socs6 / Shh
BP	GO:0018108	peptidyl-tyrosine phosphorylation	319/23210	0.000634	0.00583	Fgf7 / Socs1 / Wee1 / Itk
BP	GO:0043372	positive regulation of CD4-positive, alpha-beta T cell differentiation	30/23210	0.000642	0.00584	Myb / Socs1
BP	GO:0018212	peptidyl-tyrosine modification	322/23210	0.000657	0.00591	Fgf7 / Socs1 / Wee1 / Itk
BP	GO:0046486	glycerolipid metabolic process	325/23210	0.00068	0.00604	Fgf7 / Inpp5d / Socs1 / Socs6

BP	GO:0045580	regulation of T cell differentiation	150/23210	0.000855	0.0064	Myb / Socs1 / Shh
BP	GO:0006644	phospholipid metabolic process	348/23210	0.000877	0.00651	Fgf7 / Inpp5d / Socs1 / Socs6
BP	GO:2000516	positive regulation of CD4-positive, alpha-beta T cell activation	36/23210	0.000925	0.00681	Myb / Socs1
BP	GO:1903707	negative regulation of hemopoiesis	158/23210	0.000993	0.00708	Inpp5d / Socs1 / Shh
BP	GO:0046854	phosphatidylinositol phosphorylation	38/23210	0.001031	0.00721	Socs1 / Socs6
BP	GO:0046627	negative regulation of insulin receptor signaling pathway	41/23210	0.001199	0.00812	Socs1 / Rela
BP	GO:0046636	negative regulation of alpha-beta T cell activation	42/23210	0.001258	0.00845	Socs1 / Shh
BP	GO:0051251	positive regulation of lymphocyte activation	394/23210	0.001388	0.00877	Inpp5d / Myb / Socs1 / Shh
BP	GO:1900077	negative regulation of cellular response to insulin stimulus	45/23210	0.001443	0.00885	Socs1 / Rela
BP	GO:0045581	negative regulation of T cell differentiation	47/23210	0.001574	0.00938	Socs1 / Shh
BP	GO:0022408	negative regulation of cell-cell adhesion	187/23210	0.001612	0.00946	Socs1 / Socs6 / Shh
BP	GO:0050870	positive regulation of T cell activation	187/23210	0.001612	0.00946	Myb / Socs1 / Shh
BP	GO:0045785	positive regulation of cell adhesion	411/23210	0.001621	0.00946	Myb / Socs1 / Rela / Shh
BP	GO:0046834	lipid phosphorylation	49/23210	0.001709	0.00984	Socs1 / Socs6
BP	GO:0043370	regulation of CD4-positive, alpha-beta T cell differentiation	50/23210	0.001779	0.01011	Myb / Socs1
BP	GO:0002696	positive regulation of leukocyte activation	446/23210	0.002183	0.0112	Inpp5d / Myb / Socs1 / Shh

BP	GO:0045620	negative regulation of lymphocyte differentiation	56/23210	0.002226	0.01122	Socs1 / Shh
BP	GO:0050867	positive regulation of cell activation	461/23210	0.002461	0.01187	Inpp5d / Myb / Socs1 / Shh
BP	GO:0030258	lipid modification	218/23210	0.002494	0.01192	Inpp5d / Socs1 / Socs6
CC	GO:0005942	phosphatidylinositol 3-kinase complex	28/23436	0.000548	0.01228	Socs1 / Socs6
CC	GO:0019898	extrinsic component of membrane	314/23436	0.000576	0.01228	Picalm / Socs1 / Socs6 / Gnas
BP	GO:0002683	negative regulation of immune system process	473/23210	0.002701	0.01263	Inpp5d / Socs1 / Socs6 / Shh
BP	GO:2000514	regulation of CD4-positive, alpha-beta T cell activation	64/23210	0.002896	0.01335	Myb / Socs1
BP	GO:0046626	regulation of insulin receptor signaling pathway	75/23210	0.003953	0.01656	Socs1 / Rela
BP	GO:0007162	negative regulation of cell adhesion	284/23210	0.005235	0.01926	Socs1 / Socs6 / Shh
BP	GO:0045088	regulation of innate immune response	289/23210	0.005494	0.01971	Socs1 / Ikbke / Rela
BP	GO:1900076	regulation of cellular response to insulin stimulus	89/23210	0.005519	0.01971	Socs1 / Rela
BP	GO:0008286	insulin receptor signaling pathway	121/23210	0.009982	0.03028	Socs1 / Rela
BP	GO:0042532	negative regulation of tyrosine phosphorylation of STAT protein	14/23210	0.017356	0.03468	Socs1
BP	GO:0042976	activation of Janus kinase activity	14/23210	0.017356	0.03468	Socs1
BP	GO:0045591	positive regulation of regulatory T cell differentiation	14/23210	0.017356	0.03468	Socs1
BP	GO:0060330	regulation of response to interferon-gamma	15/23210	0.018584	0.0348	Socs1

BP	GO:0060334	regulation of interferon-gamma-mediated signaling pathway	15/23210	0.018584	0.0348	Socs1
MF	GO:0019207	kinase regulator activity	194/22710	0.026998	0.03547	Socs1 / Socs6
BP	GO:2001185	regulation of CD8-positive, alpha-beta T cell activation	17/23210	0.021037	0.03568	Socs1
BP	GO:0071407	cellular response to organic cyclic compound	482/23210	0.021796	0.03626	Socs1 / Smarca4 / Gnas
BP	GO:0050708	regulation of protein secretion	490/23210	0.022751	0.03687	Socs1 / Rab34 / Hif1a
BP	GO:0032869	cellular response to insulin stimulus	193/23210	0.024105	0.03757	Socs1 / Rela
BP	GO:0060333	interferon-gamma-mediated signaling pathway	21/23210	0.025924	0.03849	Socs1
BP	GO:0046426	negative regulation of JAK-STAT cascade	25/23210	0.030788	0.04169	Socs1
BP	GO:0032868	response to insulin	225/23210	0.031992	0.04225	Socs1 / Rela
BP	GO:1904893	negative regulation of STAT cascade	26/23210	0.032001	0.04225	Socs1
BP	GO:0045589	regulation of regulatory T cell differentiation	28/23210	0.034421	0.04329	Socs1
BP	GO:0071375	cellular response to peptide hormone stimulus	244/23210	0.037094	0.04498	Socs1 / Rela
BP	GO:0045066	regulatory T cell differentiation	31/23210	0.03804	0.04515	Socs1
BP	GO:0032147	activation of protein kinase activity	248/23210	0.038206	0.04529	Socs1 / Map3k14
BP	GO:0050730	regulation of peptidyl-tyrosine phosphorylation	263/23210	0.042488	0.04886	Fgf7 / Socs1
